# Recognition of multi-modal fusion images with irregular interference

**DOI:** 10.7717/peerj-cs.1018

**Published:** 2022-06-24

**Authors:** Yawei Wang, Yifei Chen, Dongfeng Wang

**Affiliations:** 1 College of Information and Electrical Engineering, China Agricultural University, Beijing, Beijing, China; 2 Engineering Practice Innovation Center, China Agricultural University, Beijing, Beijing, China; 3 Digital Health Division, KingSoft Cloud, Beijing, Beijing, China

**Keywords:** Objects recognition, Neural network, Computer vision, Multimodal fusion

## Abstract

Recognizing tomatoes fruits based on color images faces two problems: tomato plants have a long fruit bearing period, the colors of fruits on the same plant are different; the growth of tomato plants generally has the problem of occlusion. In this article, we proposed a neural network classification technology to detect maturity (green, orange, red) and occlusion degree for automatic picking function. The depth images (geometric boundary information) information of the fruits were integrated to the original color images (visual boundary information) to facilitate the RGB and depth information fusion into an integrated set of compact features, named RD-SSD, the *m*AP performance of RD-SSD model in maturity and occlusion degree respectively reached 0.9147.

## Introduction

In our daily life, vegetables are intricately linked to people’s lifestyle and health, so the yield and quality of vegetables are closely linked to human life. The growth of fruits makes the dynamics tough to estimate and forecast in the natural environment with the common problems of overlap and obscuration, which make it hard to evaluate the phenotype of fruits.

Recognizing tomatoes based on color images faces two problems: first, tomato plants have a long fruit bearing period, the colors of fruits on the same plant are different, and green fruits are similar to the background color of plants; second, the growth of tomato plants generally has the problem of occlusion. In order to solve the influence of the environment and fruit growth stage on the accuracy of tomatoes recognition, we propose a color and depth image fusion method to enhance the recognition accuracy for tomato fruits.

## Related work

The tomato plant has a long growth cycle, with green immature tomatoes comparable to the plant backgrounds. The intensive growth of tomato plants has the problems of occlusion, overlap and insufficient light, which brings challenges to recognition (Arefi et al., 2011; Baltazar, Aranda & Aguilar, 2008). Color thresholds are often used to segment tomatoes, for example, Khoshroo, Arefi & Khodaei (2014) based on R-G component, Junhui et al. (2021) based on R color component, Malik et al. (2018) and Uramoto et al. (2021) based on HSV color space information to detect mature tomatoes. Color space threshold can only identify a single color’s fruit, but cannot classify mature and immature tomatoes at the same time.

Recent methods focus on overcoming the problems by proposing different schemes for classifying tomato fruit maturity based on neural network (Liu, Pi & Xia, 2020; Hsieh et al., 2021). Wan et al. (2018) proposed a tomato maturity (green, orange, red) detection method that combined the characteristic color value with neural network classification technology. On the basis of the Faster R-CNN algorithm on the ResNet-50 backbone network, Sun et al. (2018) developed k-means clustering to fit the anchor frame size of the dataset and identify tomatoes’ maturity. Fonteijn et al. (2021) and Afonso et al. (2020) classified tomato maturity based on the Mask R-CNN method. Intuitively, recognition of fruit ripeness by single color images is frequently affected by the complex growing background of fruit. There is a further problem of being surrounded by obstacles, such as branches and leaves, which is one of the primary challenges for fruit recognition systems.

Multi-modal fusion technology can integrate various information extracted from different unimodal performance sources into a single compact representation, enhance the complementarity of information, and improve the detection efficiency (Gené-Mola et al., 2019). Fusion method is a key in multi-modal studies. Generally, visible light images are combined with depth maps, infrared maps to obtain compact multi-modal features. Min, Chishe & Dahui (2021) proposed a pedestrian detection algorithm based on the SSD network, which separately extracted the features of visible light and infrared images to fuse the two modalities on a multi-scale feature layer. Tao (2020) proposed a waste detection method based on vector machine classifiers with multi-scale fusion of color and depth images, and the segmentation rate of this algorithm reached 76.38%. Sa et al. (2016) fused RGB and NIR multi-modal information by the Faster R-CNN method to detect various fruits, and the F1 score was 0.83. Qian, Jiting & Jianguo (2020) proposed the up-sampling fusion method of color and depth images based on residual network, which enhanced the complementarity of information and the recognition accuracy reached 0.879. Zheng, Li & Jun (2021) extracted the multi-scale features of color and infrared images fusion based on YOLO architecture to detect pedestrians, and the AP rate was 92.6%.

**Figure 1:**
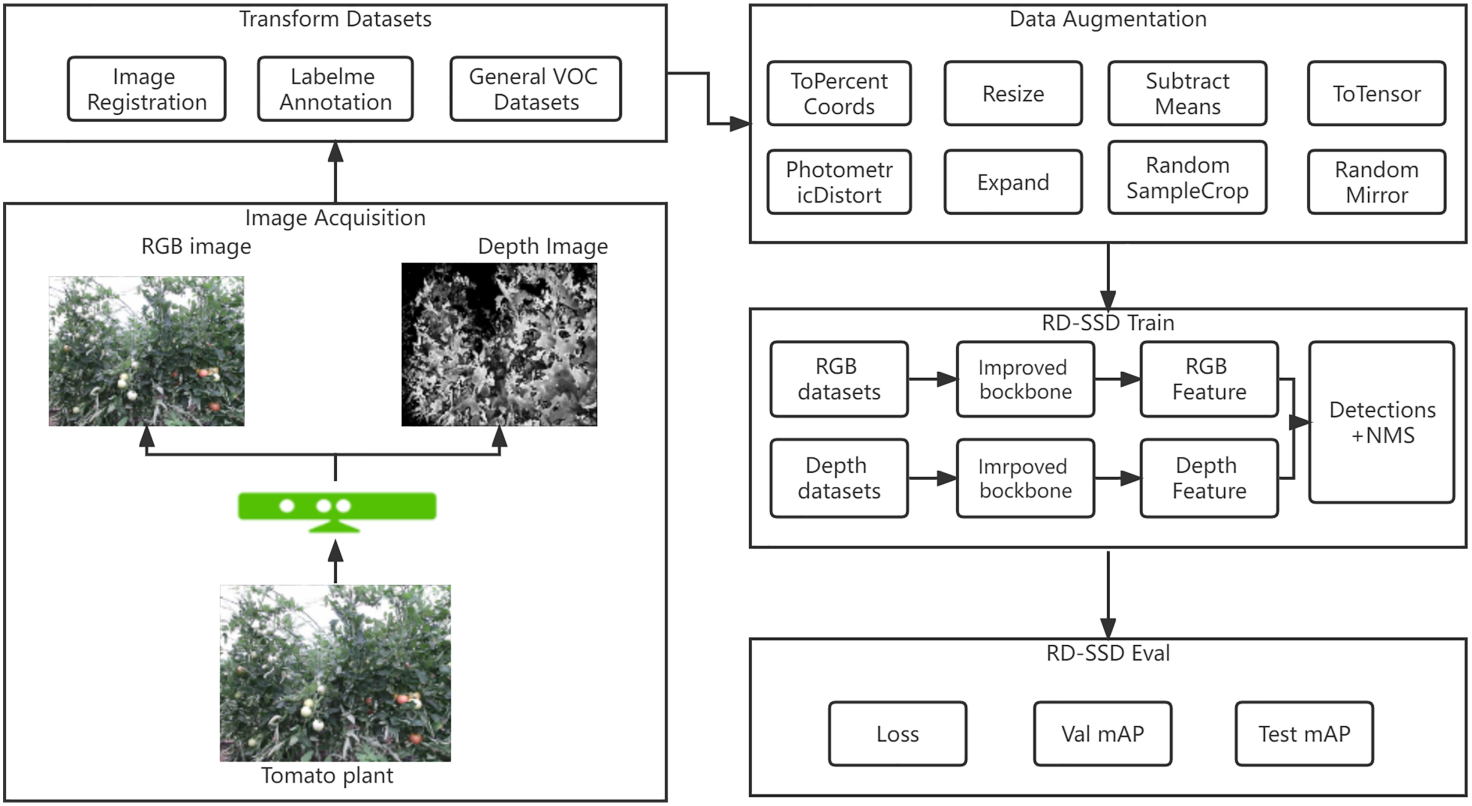
Diagram of tomato fruit recognition based on RD-SSD model.

The RGB image contains color, texture information, but the depth image contains geometric information that is more robust with lighting variations. In order to solve the influence of the environment and fruit growth stage on the accuracy of tomatoes recognition, this article proposes an RD-SSD model based on the fusion of color and depth images. Figure 1 shows the architecture of the RD-SSD model, which includes image acquisition, data annotation, image augmentation, model training and model evaluation.

### Dataset augmentation and processing

The camera used in our experiment is Kinect V2.0, which can collect RGB-D images. The size of the color image is 1,920 * 1,080 pixel, and the size of the depth image is 512 * 424 pixel. In order to achieve the fusion of color and depth images, it is necessary to unify the size of the color and depth images with Windows SDK (Kinect SDK, 2014). The approach to detecting tomatoes target is bounding selection method, and one classification standard is the occlusion of fruit (occlusion or non-occlusion), and the other one is maturity degree (green, orange, and red) for combining six types of objects, where $1\_i$ represents non-occluded immature fruits, $2\_i$ represents occluded immature fruits, $3\_i$ represents non-occluded semi-mature fruits, $4\_i$ represents occluded semi-mature fruits, $5\_i$ represents non-occluded mature fruits, and $6\_i$ represents occluded mature fruits.

**Figure 2:**
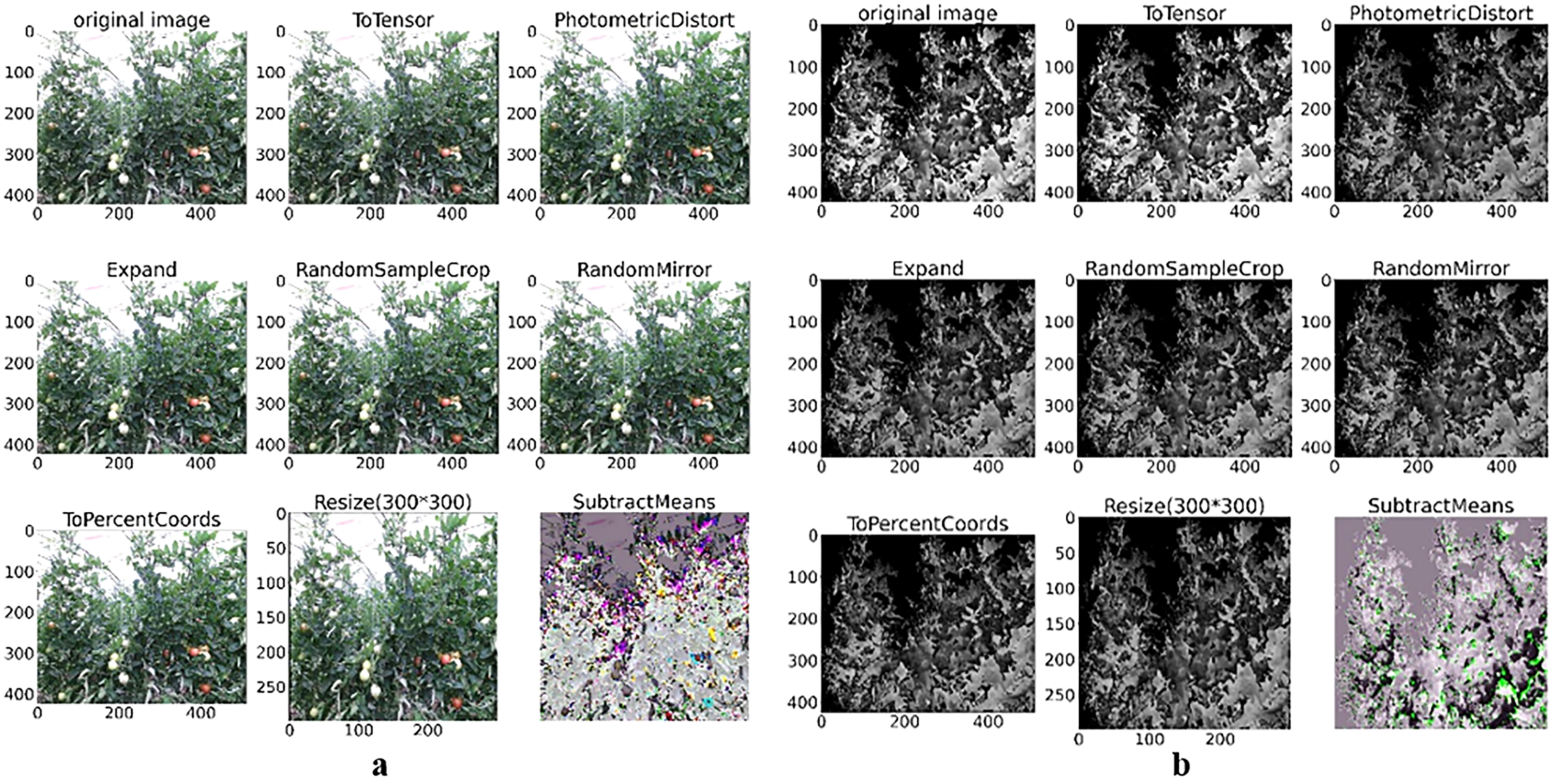
Color and depth image data augmentation of tomato plant. (A) Color image augmentation methods and (B) depth image augmentation methods.

Each image in the training set is augmented with eight transformed versions by ToSensor, PhotometricDistort, Expand, RandomSampleCrop, RandomMirror, ToPercentCoords, Resize, and the SubtractMeans method in Fig. 2, which perform random translation, scaling, rotation and color transformation.

Figure 2A depicts the color image data augmentation transformation results, and Fig. 2B shows the depth image data augmentation transformation results.

**Figure 3:**
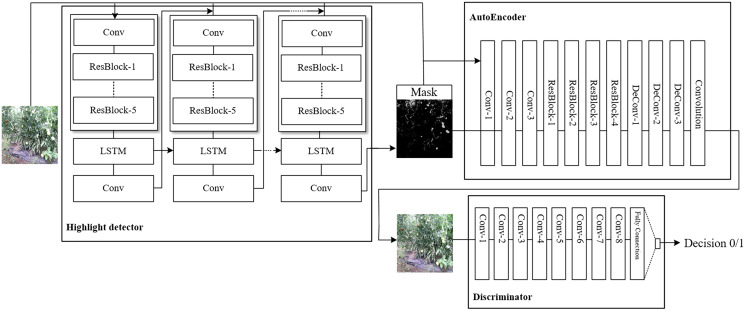
Framework overview of the proposed HRGAN.

In the tomato growing environments, obtaining images is susceptible of illumination that affect the identification accuracy. To address the problem, we propose a novel method HRGAN to learn removing highlight from unpaired training data, and learn the potential relationship between the highlight image domain $H$ and the highlight-free image domain $\rm F$. Train a generator network $G$, which takes highlight images ${I_h} \in H$ as input to generate highlight-free image ${I_f} \in F$. Then exploit $D$ as the discriminator to identify the generated images, as shown in Fig. 3.

For the Generator network $G$, we explored Long and Short Term Memory (LSTM) to preserve the valuable features to ensure the realistic of detected highlight regions. The LSTM contains an input gate ${i_t}$, an output gate ${o_t}$, a forget gate ${f_t}$, and a cell state ${C_t}$, where $t$ is time (Ding et al., 2019). The input of LSTM as shown in Eq. (1):

${i_t} = \sigma \left( {{W_{xi}}{X_t} + {W_{hi}}{H_{t - 1}} + {W_{ci}} \odot {C_{t - 1}} + {b_i}} \right)$

${f_t} = \sigma \left( {{W_{xf}}{X_t} + {W_{hf}}{H_{t - 1}} + {W_{cf}} \odot {C_{t - 1}} + {b_f}} \right)$

${C_t} = {f_t} \odot {C_{t - 1}} + {i_t} \odot tanh \left( {{W_{xc}}{X_t} + {W_{hc}}{H_{t - 1}} + {b_c}} \right)$

${o_t} = \sigma \left( {{W_{xo}}{X_t} + {W_{ho}}{H_{t - 1}} + {W_{co}} \odot {C_t} + {b_o}} \right)$

(1) $${H_t} = {o_t} \odot tanh\left( {{C_t}} \right)$$

where ${X_t}$ is the output feature of the residual module, $W$ and $b$ is the weight and deviation respectively, ${C_t}$ indicates the cell state that will be input to the next step, ${H_t}$ represents the output feature of the LSTM unit. We initialize the value of the highlight intensity as 0.5. At each time step, the current attention map is connected to the input image into the next recursive block of the recurrent network.

The loss of HRGAN comes from the highlight detector and discriminator as Eq. (2):

(2) $${L_T} = {L_M} + {L_P} + {L_{Adv}}$$

The highlight detector compares the generated intensity mask $\left\{ M \right\}_1^N$ with the ground truth $T$. The detector is generated from $1$ to $N$ recursive blocks as in Eq. (3):

(3) $${L_M} = \mathop \sum \nolimits_{i = 1}^N {\beta _i}\cdot {L_{MSE}}\left( {{M_i},\; {T_i}} \right)$$

where ${M_i}$ is the output extracted of the $i$th layer, and ${T_i}$ is the ground truth with the same size of the $i$th layer. ${\beta _i}$ is the weights of the Mean Square Error (MSE) loss for the $i$th iteration, we design ${\beta _i} = {0.5^{N - i + 1}}$.

${L_P}$ is designed to calculate the global difference between the ground truth image and the highlight removal result (Qian et al., 2018). We extract image features by VGG16 (Simonyan & Zisserman, 2014) pretrained on ImageNet datasets, the perceptual loss as in Eq. (4):

(4) $${L_P} = {L_{MSE}}\left( {VGG\left( {{L_i}} \right),\; \; VGG\left( T \right)} \right)$$

where $VGG\left( {{L_i}} \right)$ and $VGG\left( T \right)$ are the feature of image ${L_i}$ and $T$ trained from VGG16 network. The discriminator network validates whether the image produced by the generative network looks real. The generative adversarial loss ${L_{Adv}}$ can be designed as Eq. (5):

(5) $${L_{Adv}} = {{\rm {\mathbb E}}_{{I_f}\sim pclear}}\left[ {log\left( {D\left( {{I_f}} \right)} \right)} \right] + {{\rm {\mathbb E}}_{{I_h}\sim phighlight}}\left[ {log\left( {1 - D\left( {G\left( {{I_h}} \right)} \right)} \right)} \right]$$

### Refinement backbone based on Inception module

**Figure 4:**
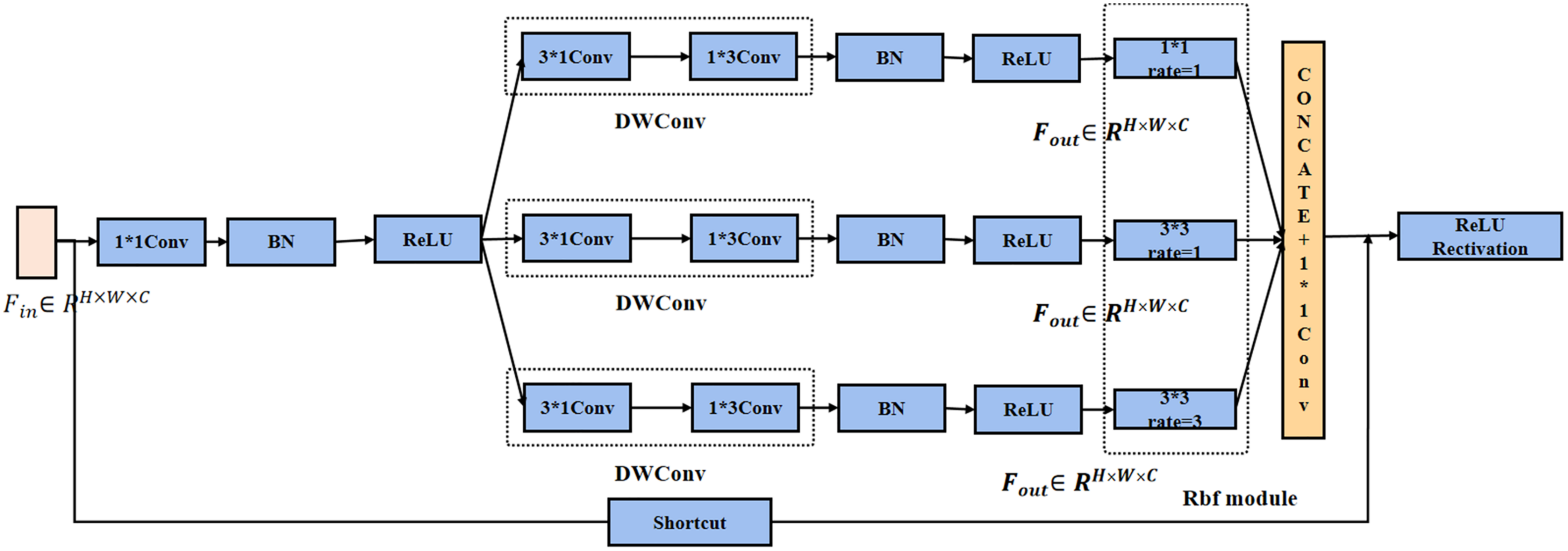
Improved of Inception model for recognition.

Since AlexNet (Krizhevsky, Sutskever & Hinton, 2012), increasingly deeper networks have been proposed to solve more complex problems, such as VGG16, VGG19, and GoogleNet (Szegedy et al., 2014). In this study, a modified version of multi-scale Inception block was designed for the problems of GoogleNet and the characteristics of recognition. This network draws on the main architecture of Inception v1 block. Multi-scale network structure is shown in Fig. 4, and the following improvements are made in two aspects:

The Inception network structure adopts two methods for convolution kernel decomposition. 1 * 3 with 3 * 1 convolution kernels was used instead of 3 * 3 kernel for feature maps. This method can ensure that the receptive field of the decomposed convolution kernel not be changed.

Each branch corresponds to a different size of the receptive fields (RF) (Liu, Huang & Wang, 2018), using the expansion convolution to control their eccentricity, and its dimensions are adjusted to generate the final feature map. For the same case of kernel 7 * 7, the conventional convolution can only obtain 5 * 5 receptive fields after 3 * 3 convolution kernel processing. The receptive field of 7 * 7 can be obtained after 3 * 3 convolution kernel with dilation rate of 2.

### Development recognition model based on SSD algorithm

Multi-modal images used in this article, color images and depth images are denoted as ${I_{rgb}}$ and ${I_{depth}}$ respectively. The shape of ${I_{rgb}}$ is 3 * 512 * 424, and the shape of ${I_{depth}}$ is 1 * 512 * 424. After data augmentation, the image size is scaled to 3 * 300 * 300 and 1 * 300 * 300 as input layers to provide a basis for generating features. The feature map generation network is designed separately for the color and depth images to extract feature maps from different stages. Multi-scale feature maps are the results of convolutions, which express different levels of images’ information, such as local features, edge features, texture features, and so on. The feature maps generated by color and depth image through the network are recorded as ${C_r}\left( n \right)$ and ${C_d}\left( n \right)$, as shown in Eq. (6):

${C_r}\left( n \right) = f\left( {{C_r}\left( {n - 1} \right)} \right),{\rm \; }{C_r}\left( 0 \right) = {I_{rgb}}$

(6) $${C_d}\left( n \right) = f\left( {{C_d}\left( {n - 1} \right)} \right),{C_d}\left( 0 \right) = {I_{depth}}$$

where $r$ represents color image, $d$ represents depth image, $n$ represents different feature layers, and $f\;$ represents convolution and pooling operations on the feature layers. The sizes of six types of feature maps in color and depth image are 38 * 38, 19 * 19, 10 * 10, 5 * 5, 3 * 3 and 1 * 1. Furthermore, as high-level features have larger receptive fields and capture more semantic information, low-level features have higher resolution and contain accurate localization details, which are complementary to abstract features. The characteristic maps of each layer are combined to obtain the color and depth characteristic map set that cover the fruit characteristics from multiple scale receptive fields, which are denoted as ${F_{rgb}}$ and ${F_{depth}}$, as shown in Eq. (7):

${F_{rgb}} = {\rm U}_{i = s1}^s{C_r}\left( i \right)$

(7) $${F_{depth}} = {\rm U}_{i = s1}^s{C_d}\left( i \right)$$

**Figure 5:**
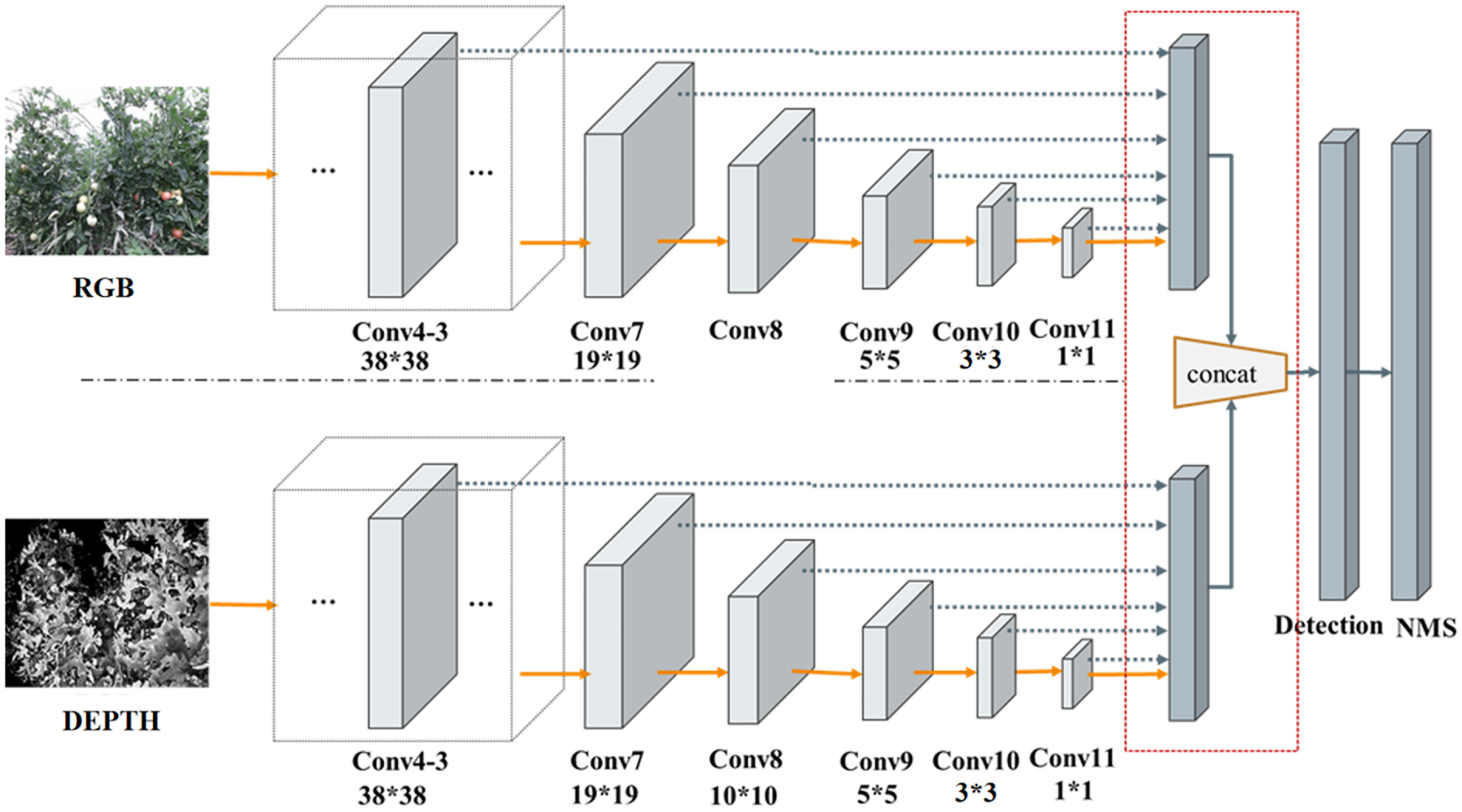
Subnetworks architecture of the RD-SSD model.

RD-SSD architecture is composed of two parallel subnetworks, RGB-Network and Depth-Network, both of which form a neural network. Figure 5 shows the characteristic network diagram of RD-SSD model, including six color feature maps (conv4_3-r, conv7(FC7)-r, conv8-r, conv9-r, conv10- r, conv11-r) and six depth feature maps (conv4_3-d, conv7(FC7)-d, conv8-d, conv9-d, conv10-d, conv11-d).

The resulting prior boxes on the feature maps are then fed to the detection network to produce the result on the reference layers. On the outputs of the detection layer, each image generates six feature maps for ${n^2}$ center points, and each center point generates $k$ prior boxes. For color image and depth image fusion methods, conv4_3-r, conv7(FC7)-r, conv8-r, conv9-r, conv10-r, conv11-r, conv4_3-d, conv7(FC7)-d, conv8- d, conv9-d, conv10-d, conv11-d feature layers set (4, 6, 6, 6, 4, 4, 4, 6, 6, 6, 4, 4) prior boxes respectively. For each feature point of the feature map, we assign the corresponding prior boxes to the feature map layers. ${B_{rgb}}$ and ${B_{depth}}$ are fully connected to generate the sum of a prior boxes as ${B_{all}}$. The calculation formula is shown in Eq. (8):

(8) $${B_{all}}\left( {\rm n} \right) = {B_{rgb}}\left( {\rm n} \right) + {B_{depth}}\left( {\rm n} \right)$$

The size of the SSD prior box is related to the shape characteristics of the identified object, which often contains squares and rectangles of different proportions and sizes. The dimensions of multiple prior boxes should be guaranteed in the occluded fruit recognition model to improve the generalization ability to identify the fruit. The setting of the prior box includes scale and aspect ratio, which is calculated as Eq. (9):

(9) $${s_k} = {s_{min}} + \displaystyle{{{s_{max}} - {s_{min}}} \over {m - 1}}\left( {k - 1} \right),\;\;\;\ k \in \left[ {1,m} \right]$$

$m$ is the number of feature maps, ${s_k}$ is the relative ratio of the prior box to the feature map, ${s_{min}}$ is 0.2 and ${s_{max}}$ is 0.9.

For the position of the a prior frame, set the center of the a prior frame as $\left\{ {\displaystyle{{\alpha + 0.5} \over {\left| {{f_k}} \right|}},\; \displaystyle{{b + 0.5} \over {\left| {{f_k}} \right|}}} \right\}$, where $\left| {{f_k}} \right|$ is the size of the $k$ characteristic graph, a, b ∈ $\left\{ {0,1,2,{\rm \; }\left| {{f_k} - 1} \right|} \right\}$ and normalize the coordinates of the a prior frame to make it within 0,1. The mapping relationship between the a prior frame coordinates on the feature map and the original image coordinates as Eq. (10):

${x_{min}} = \left\{ {\displaystyle{{b + 0.5} \over {\left| {{f_k}} \right|}} - \displaystyle{{{w_k}} \over 2}} \right\}\displaystyle{{{w_{img}}} \over {{w_{feature}}}},\;{y_{min}} = \left\{ {\displaystyle{{b + 0.5} \over {\left| {{f_k}} \right|}} - \displaystyle{{{h_k}} \over 2}} \right\}\displaystyle{{{h_{img}}} \over {{h_{feature}}}}$

(10) $${x_{max}} = \left\{ {\displaystyle{{\alpha + 0.5} \over {\left| {{f_k}} \right|}} + \displaystyle{{{w_k}} \over 2}} \right\}\displaystyle{{{w_{img}}} \over {{w_{feature}}}},\; {y_{max}} = \left\{ {\displaystyle{{\alpha + 0.5} \over {\left| {{f_k}} \right|}} + \displaystyle{{{h_k}} \over 2}} \right\}\displaystyle{{{h_{img}}} \over {{h_{feature}}}}$$

${w_{feature}}$ and ${h_{feature}}$ is the width and height of the feature layer, ${w_{img}}$ and ${h_{img}}$ are the width and height of the original image, and the obtained ( ${x_{min}}$, ${y_{min}}$, ${x_{max}}$, ${y_{max}}$) is the coordinate mapped to the original image by the a prior frame with the center of $\left\{ {\displaystyle{{\alpha + 0.5} \over {\left| {{f_k}} \right|}},\; \displaystyle{{b + 0.5} \over {\left| {{f_k}} \right|}}} \right\}$ and the size of $\left( {{w_k},\; {h_k}} \right)$ on the feature graph of layer $k$.

In the natural growth of tomatoes, there are occlusions by leaves, stems and fruits, and the scale setting of boxes is related to the occlusion, with the aspect ratio ${{\rm a}_{\rm r}} \in \left\{ {1,2,3,\; \displaystyle{1 \over 2},\displaystyle{1 \over 3}} \right\}$. 1 means the aspect ratio is 1:1, 2 means the aspect ratio is 2:1, 3 means the aspect ratio is 3:1, $\displaystyle{1 \over 2}$ means the aspect ratio is 1:2, $\displaystyle{1 \over 3}$ means the aspect ratio is 1:3. As shown in Table 1, we used different scales and aspect ratios parameters for feature maps. In addition, with the deeper of the feature map increases, the receptive field becomes larger.

**Table 1:** A prior box calculation result for object recognition.

Feature map	Size	s_min (s_k)	s_max (s_(k+1))	a_r
1	38 * 38	30	60	1, 2
2	19 * 19	60	111	1, 2, 3
3	10 * 10	111	162	1, 2, 3
4	5 * 5	162	213	1, 2, 3
5	3 * 3	213	264	1, 2
6	1 * 1	264	315	1, 2

RGB-D target recognition methods based on early fusion methods can take advantage of the correlation between multiple features from different patterns in the early stage, which helps to better complete the task (Snoek, Worring & Smeulders, 2005). However, the decisions level usually have the same representation, which makes decision fusion easier (Gao et al., 2019).

RD-SSD designs the overlap maximization between prior boxes of feature map with the real target $P$, which measures the overlap between ground truth boundaries and forecast boundaries for real target. The formula of IoU for all prior boxes is demonstrated in Eq. (11):

(11) $$Io{U_{all}} = \displaystyle{{{B_{al{l_i}}} \cap {P_j}} \over {{B_{al{l_i}}} \cup {P_j}}}\; \; {\rm \; }i \in \left[ {0,num\left( {{B_{all}}} \right)\left] {,j \in\ } \right[0,num\left( P \right)} \right]$$

where $i$ is the number of the priori box, $j$ is the number of ground truth of fruit objects. In this article, a prior box to be true only if IoU of the prior box ${B_{all}}$ with the ground truth bounding box is greater than 0.5. A large number of default bounding boxes can be generated after sampling and grouping on the same feature points. In the post processing stage of fruit detection, NMS is commonly used to filter the generated boxes. Lastly, an optimal bounding box is reserved for the same fruit to eliminate overlapping prior boxes, the process of IoU bounding box losses is shown in Table 2.

**Table 2:** Implement the model of IoU bounding box losses for RD-SSD model.

Algorithm 1: *IoU* as bounding box losses
Input: Predicted ${{\rm B}_{al{l_i}}}$ and ground truth ${P_j}$ bounding box
Coordinates:
${B_{\rm i}} = \left( {x_{min}^b,\; y_{min}^b,\; x_{max}^b,\; y_{max}^b} \right),$ ${P_j} = \left( {x_{min}^p,\; y_{min}^p,\; x_{max}^p,\; y_{max}^p} \right)$
Output: $Io{U_{all}}$
For the predicted box $B$ and $P$
ensuring $x_{max}^r > x_{min}^r\; and\; y_{max}^r > y_{min}^r$
ensuring $x_{max}^d > x_{min}^d\; and\; y_{max}^d > y_{min}^d$
Calculating area of *B*, ${A^b}$ = $\left( {x_{max}^b - x_{min}^b} \right) \times \left( {y_{max}^b - y_{min}^b} \right)$
Calculating area of $P,\; {A^p}$ = $\left( {x_{max}^p - x_{min}^p} \right) \times \left( {y_{max}^p - y_{min}^p} \right)$
Calcuating intersection *I between B and* $P$
$I = \; \left\{ {\matrix{ {\left( {min\left( {x_{max}^b,x_{max}^p} \right) - max\left( {x_{min}^p,x_{min}^b} \right)} \right) \times \left( {min\left( {y_{max}^b,y_{max}^p} \right) - max\left( {y_{min}^p,y_{min}^b} \right)} \right)} \cr 0 \cr } } \right.$
* IoU* = $\displaystyle{I \over {{A^b} + {A^p} - I}}$
end For

The loss function of RD-SSD model consists of position loss ${L_{all\_loss}}$ and the classification confidence loss ${C_{loss}}$ (Liu et al., 2016). The comprehensive loss ${F_{loss}}$ is the weighted value of the position loss and the confidence loss. The calculation formula is shown in Eq. (12):

(12) $$\; {F_{loss}}\left( {t,c,l,g} \right) = \displaystyle{1 \over N}\left( {{C_{loss}}\left( {t,c} \right){\rm \; } + {\rm \; }{L_{all\_loss}}\left( {t,l,g} \right)} \right)$$

where $l$ is the position of the target, and $c$ is the classification.

## Experiments

### Data and experiment setup

PyTorch deep learning framework is used in this article. The computing resource for the deep learning experiment is CPU2678 v3 * 2 (24 cores and 48 threads), 16G memory, GTX 1080ti 11G graphics card, and Ubuntu 18.6 operating system.

The dataset contains color and depth images, divided into 64% training data, 16% validation data, and 20% test data. The datasets include six categories, the first setup corresponds to the non-occluded and occluded scene, whereas the second setup corresponds to the maturity of fruits, non-occluded immature tomatoes as tomato1, occluded immature tomatoes as tomato2, non-occluded semi-mature tomatoes as tomato3, occluded semi-mature tomatoes as tomato4, non-occluded mature tomatoes as tomato5, occluded mature tomatoes as tomato6. The statistics about each dataset are shown in Table 3.

**Table 3:** Statistics for tomato fruits images.

Label	Number of fruits	Meaning
Tomato1	3,914	non-occluded immature tomatoes
Tomato2	3,132	occluded immature tomatoes
Tomato3	2,209	non-occluded semimature tomatoes
Tomato4	3,317	occluded immature tomatoes
Tomato5	1,313	non-occluded mature tomatoes
Tomato6	2,031	occluded mature tomatoes

### Experiment based on color image

The color map is extracted from specified convolution layers, and 8,732 prior boxes are obtained. The localization and classification of tomato fruit in the natural scene are accepted through the basic network architecture. During model training, batch_size is set to 8, iteration is set to 120,000, Learning rate (*lr*) is set to 1e−3, and test set evaluation is carried out every 500 iterations.

**Figure 6:**
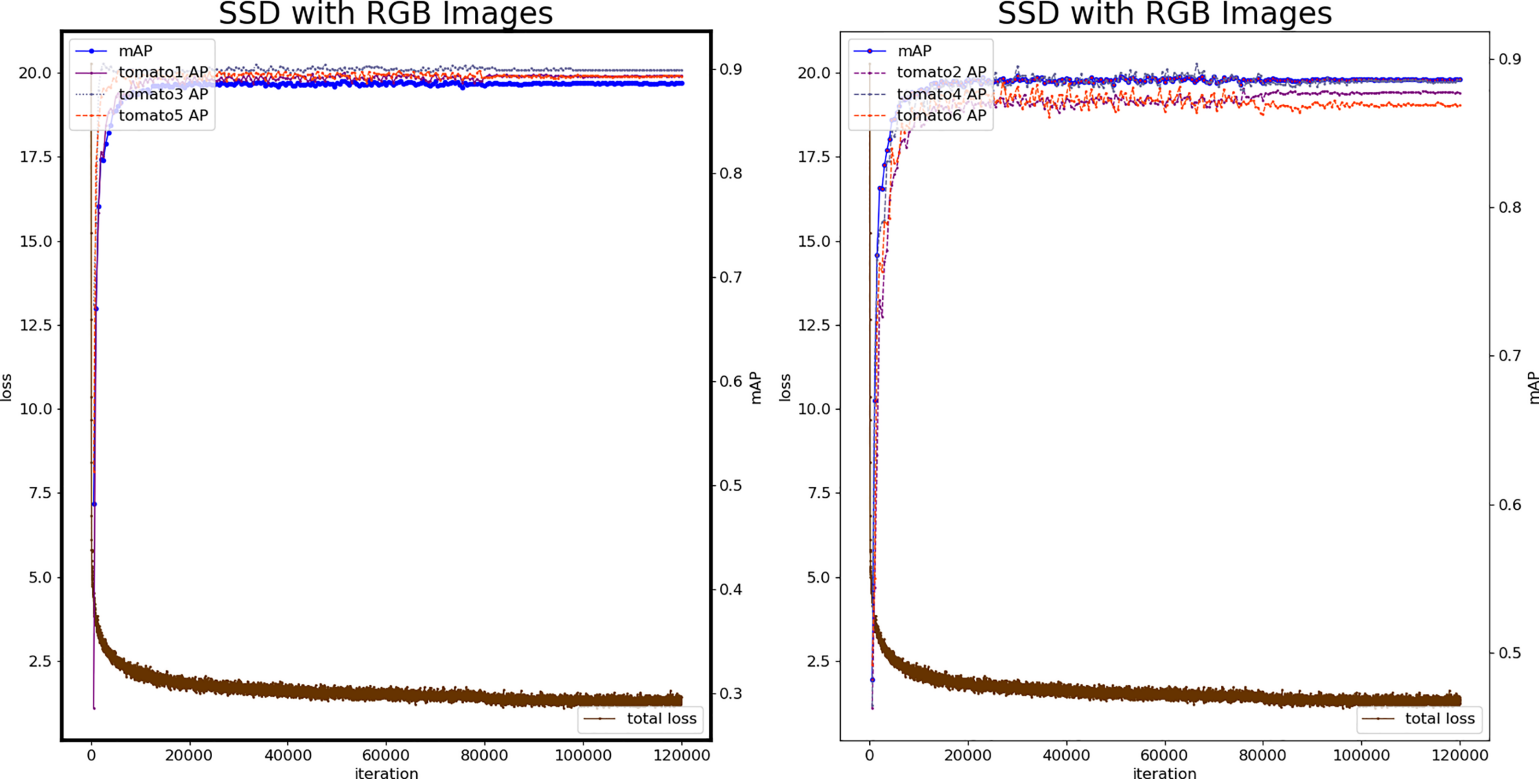
Loss and mAP changes of the color-SSD model.

Figure 6 shows the loss plots and mAP (six categories) during the training procedure. The 26,000^th^ iteration model is the best, mAP reaches 0.8914, and the loss value is 1.688, which is at the same level as the minimum loss value. Therefore, the model produced by 26,000 iterations is used as the color-SSD tomato fruit classification model.

### Experiment based on depth image

The SSD model is constructed based on the depth image to verify the recognition effect of the model. Similar to the SSD operation process of the color image, the SSD network is used to extract the features of depth map, and obtain 8,732 prior boxes.

**Figure 7:**
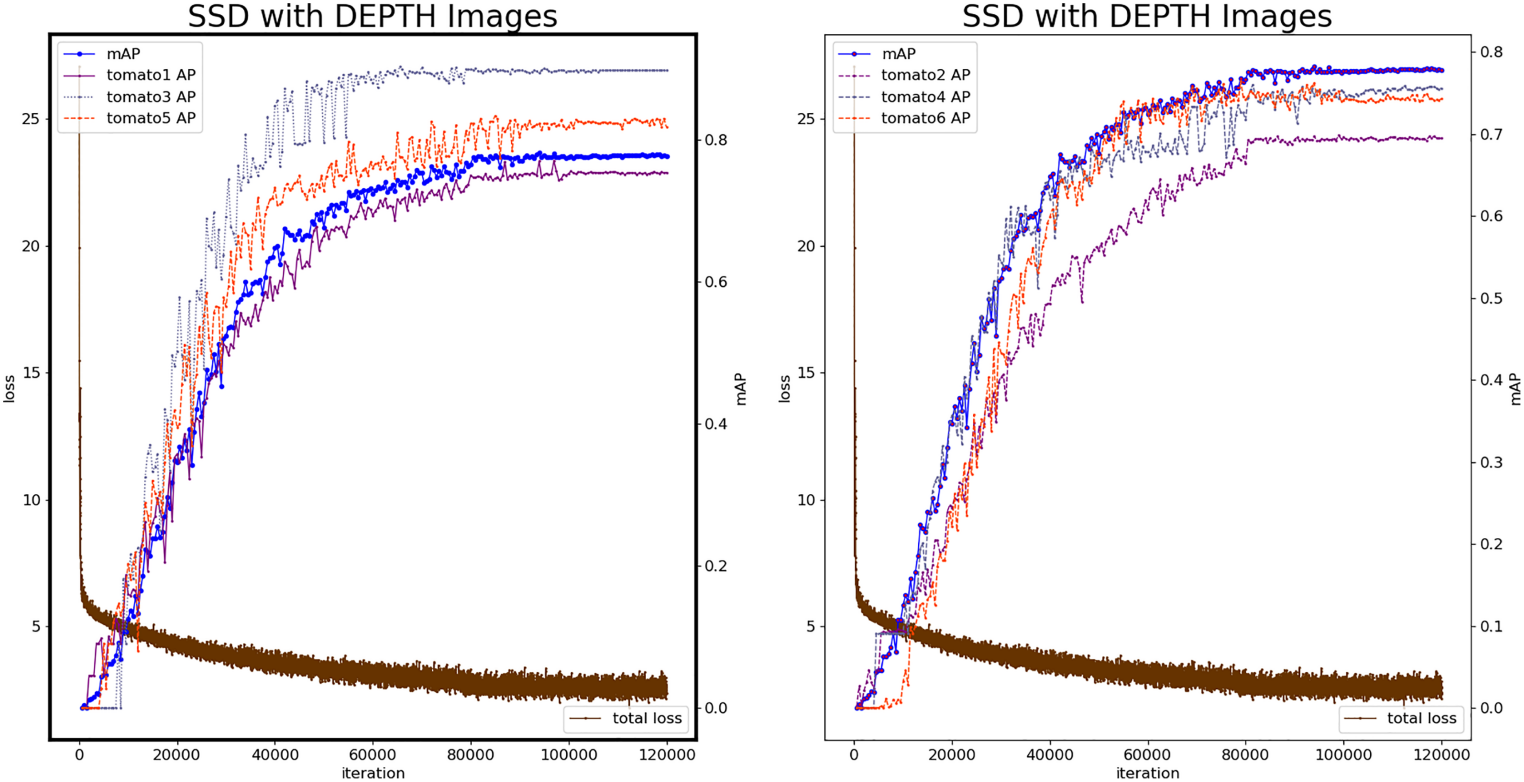
Loss and mAP changes of depth-SSD model.

Figure 7 shows the loss value and mAP change during the SSD model by depth images. At the 112,380^th^ iteration, the minimum loss value is 1.584, and the number of iterations with stable loss value is higher than color-SSD model. The 93,500^th^ iteration model was the best with mAP reaching 0.7876. The mAP of the depth-SSD recognition model is lower than the color-SSD model. The depth image reflects the position information of the fruit, during the feature learning process, which is sensitive to the edge information of the fruit, and can identify the fruit that is occluded.

### Experiment based on RD-SSD

**Figure 8:**
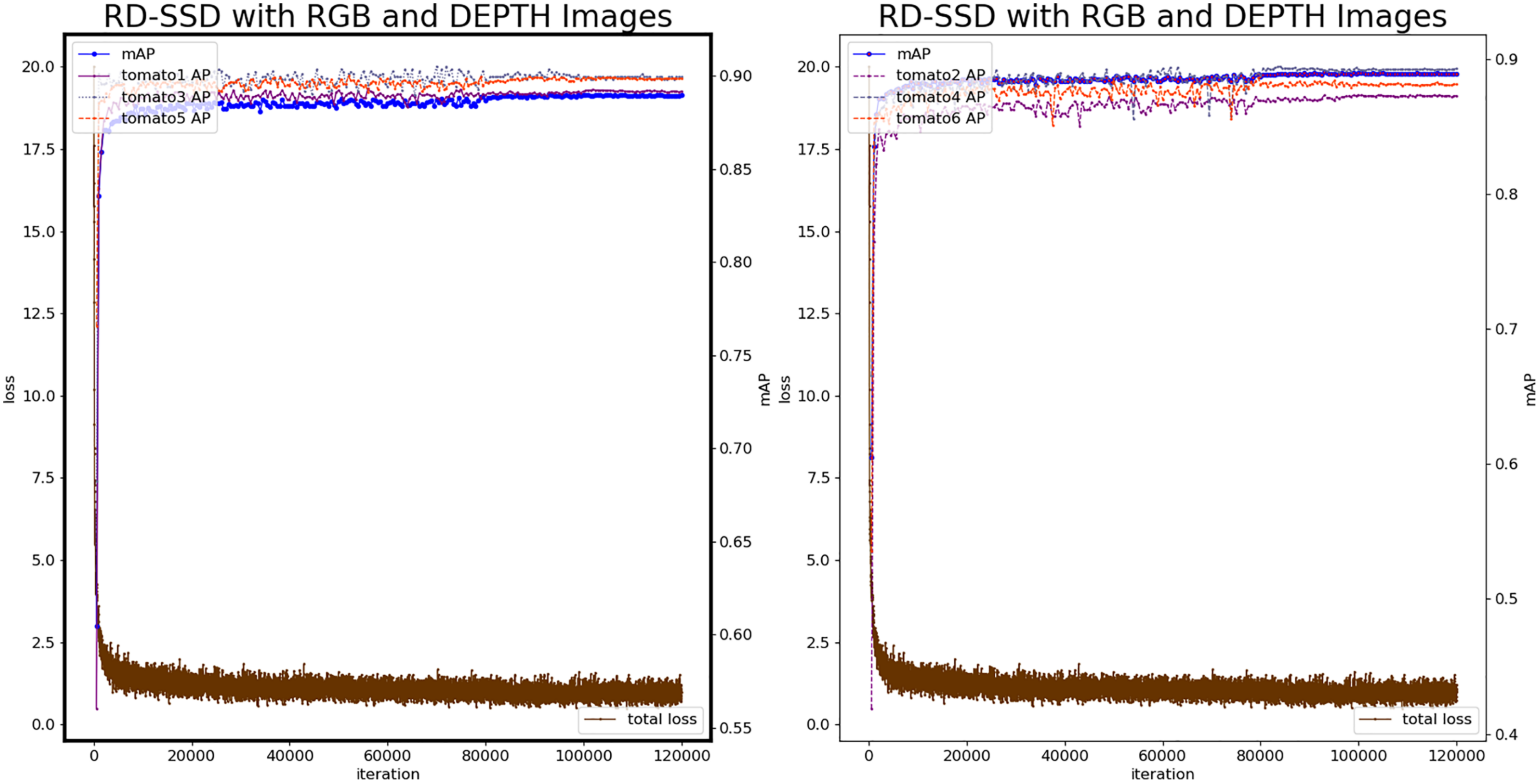
Loss and mAP changes of the RD-SSD model.

Based on the RD-SSD model, it realizes tomato fruit recognition, tomato maturity classification and occlusion classification. In the neural network, there are two branches corresponding to images from color and depth, for feature extraction. The number of prior frames ${N_{proir}}$ of the feature map after fusion is 17,464. The number of maximum iterations is 120,000, the test set is verified every 500 iterations, the learning rate is 1e−3, the batch_size is 8, the optimizer uses the Adaptive Moment Estimation (Adam) method. Figure 8 shows the loss value and mAP during the training procedure of RD-SSD model.

The analysis shows that the RD-SSD model reaches a stable state during the training procedure and the number of iterations required less than the color-SSD and depth-SSD models. The loss value is lower than the color-SSD and depth-SSD models, indicating that the recognition deviation on the verification set is smaller, and the model recognition effect is better. The model was optimal at the 92,500^th^ iteration, mAP reached 0.9147, loss value was 0.72, and the minimum loss value at the same level. The classification accuracy AP of Tomato1 is 0.9141, the AP of tomato2 is 0.9031, the AP of tomato3 is 0.9243, the AP of tomato4 is 0.9173, the AP of tomato5 is 0.9207, and the classification accuracy of tomato6 is 0.9082.

In order to compare the overall effect of the model experiment, the results of the three model methods of color-SSD, depth-SSD and RD-SSD are compared and analyzed as a whole. Table 4 shows the comparison of tomato fruit recognition and classification recognition results, including the overall model recognition effect and the recognition effect of each classification.

**Table 4:** Results of tomato fruit identification and classification.

Model	mAP	Tomato1	Tomato2	Tomato3	Tomato4	Tomato5	Tomato6
R-SSD	0.8914	0.8994	0.8786	0.9021	0.8931	0.8994	0.8758
D-SSD	0.7876	0.7684	0.7138	0.8935	0.7851	0.8219	0.7429
RD-SSD	0.9147	0.9141	0.9031	0.9243	0.9173	0.9207	0.9082

In a comparison (Tables 5 and 6) to Faster R-CNN (Ren et al., 2015), FSSD (Li & Zhou, 2017), DSSD (Fu et al., 2017), YOLO (Redmon & Farhadi, 2018) (Tables 5 and 6), RD-SSD was significantly more accurate due to the use of decision from multiple feature maps and matching strategy. The RD-SSD model combining the color and depth map feature information improves the recognition rate of occluded tomatoes with color image features mainly contribute to the classification of fruit maturity, and depth image features mainly contribute to occlusion recognition. The fusion of visible and depth images improves the perception ability of tomato fruit system in target maturity classification and occlusion recognition.

**Table 5:** Comparation of identification results of tomato fruit maturity with other methods.

Algorithm	Immature	Semimature	Mature
Faster R-CNN	0.8018	0.8312	0.8128
FSSD	0.8231	0.8543	0.8327
DSSD	0.8446	0.8709	0.8287
YOLO	0.8901	0.8879	0.8831
Ours	0.9086	0.9208	0.9145

**Table 6:** Comparation of identification results of tomato fruit occlusion with other methods.

Algorithm	mAP	Nonocclusion	Occlusion
Faster R-CNN	0.8152	0.8335	0.7970
FSSD	0.8367	0.8421	0.8313
DSSD	0.8480	0.8685	0.8276
YOLO	0.8864	0.8914	0.8814
Ours	0.9147	0.9197	0.9095

## Discussion

**Figure 9:**
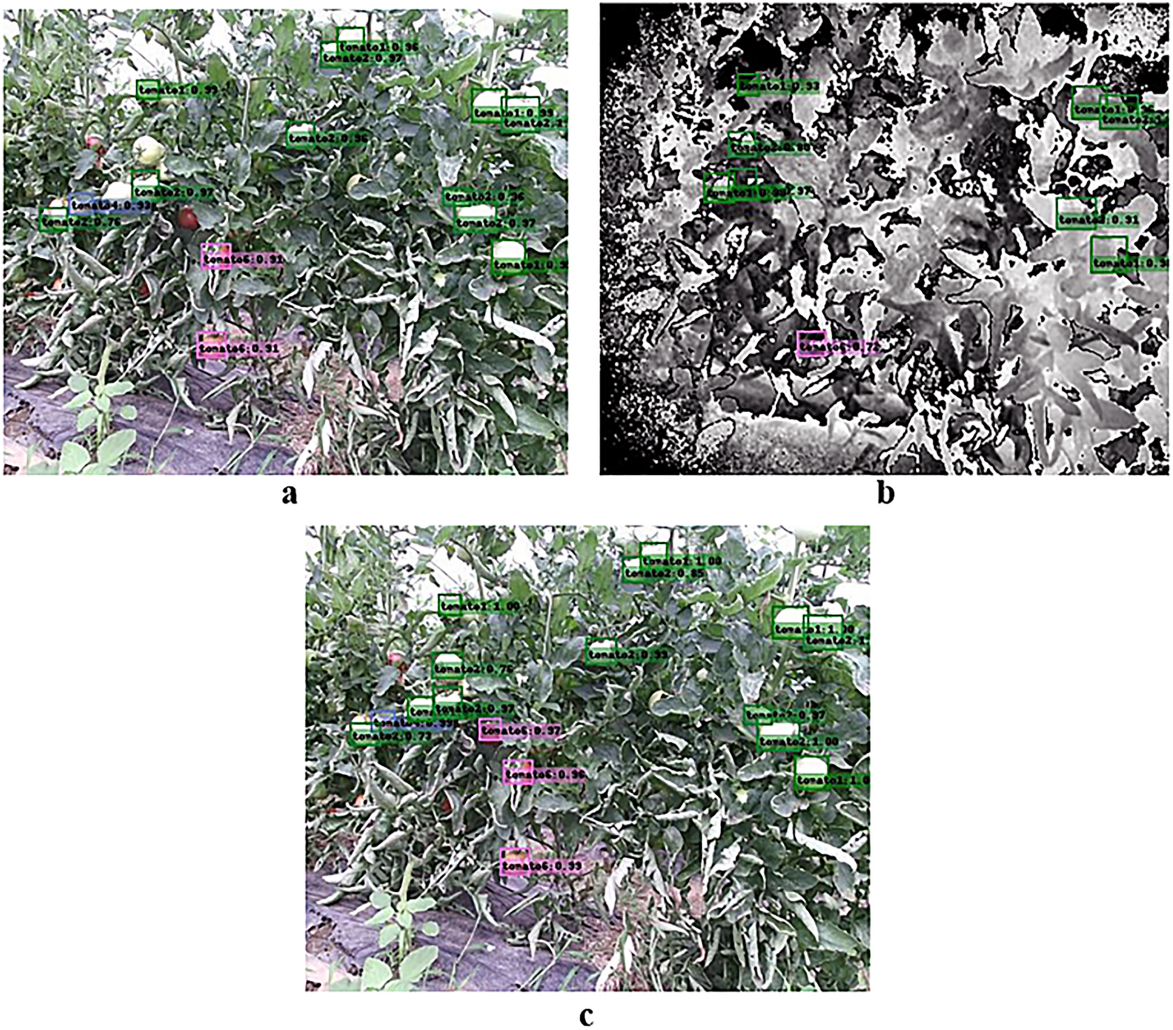
Comparison between the obtained recognition for three different models. (A) Recognition results of the color-SSD model, (B) recognition results of the depth-SSD model, and (C) recognition results of the RD-SSD model.

In order to more intuitively express the recognition effects of the color-SSD, depth-SSD and RD-SSD optimal models, this section analyzes and compares the recognition results of the test images. Figure 9 shows a comparison between the obtained recognition for three different models. The image has a complex background, with many tomato fruits and dense leaves, which is a typical complex background object recognition scene. The color-SSD model identified 14 tomatoes, including immature, semimature, mature, occluded and nonoccluded fruits; the depth-SSD model identified nine tomatoes, mainly immature tomatoes; the RD-SSD model identified 16 tomatoes with two increases relative to color-SSD, one was a ripe tomato obscured by leaves, and the other was an immature tomato overlapped with adjacent fruits. Specifically, the results showed that the RD-SSD model learned the edge information of fruit in depth image, and the recognition effect was improved compared with the other two models.

## Conclusions

In this article, to effectively integrate multi-modal features and generate accurate feature maps, a multi-modal deep aggregation module RD-SSD to facilitate the efficient fusion of texture and depth features. The plant images with different maturity and occlusion degrees were selected to construct the data set, and through data augmentation to improve the generalization ability of the model and the distinguishing degree of features.

In terms of the classification effect of tomato fruit maturity and occlusion, the recognition rate AP of the RD-SSD model for the six types of fruits reached 0.9141, 0.9031, 0.9243, 0.9173, 0.9207 and 0.9082. After adding the depth image on the basis of color image recognition, the classification effect of the occlusion of the fruit is improved. The multi-modal fusion method provides a new direction for plant fruit identification and classification, and has certain research value for the study of fruit phenotypes during fruit setting and fruiting period.

## Supplemental Information

10.7717/peerj-cs.1018/supp-1 Supplemental Information 1 The test datasets of tomato.

## Additional Information and Declarations

### Data Availability

The following information was supplied regarding data availability: The data is available in the Supplemental File.
