# Supplementary figures and images for: Recognition of multi-modal fusion images with irregular interference
Source: PeerJ Comput Sci. 2022 Jun 24;8:e1018. doi: 10.7717/peerj-cs.1018 (PMC9299258; doi:10.7717/peerj-cs.1018)

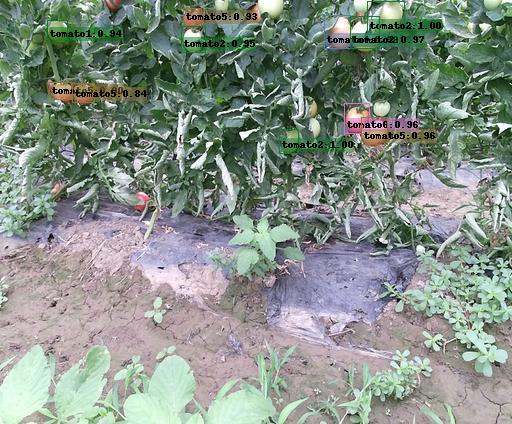

Supplement: Supplemental Information 1 [file peerj-cs-08-1018-s001.zip › data/0617.jpg]

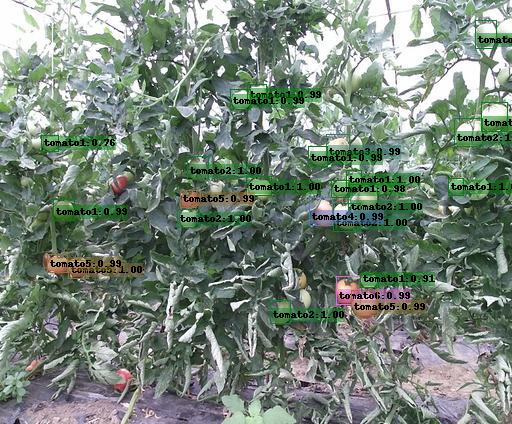

Supplement: Supplemental Information 1 [file peerj-cs-08-1018-s001.zip › data/0629.jpg]

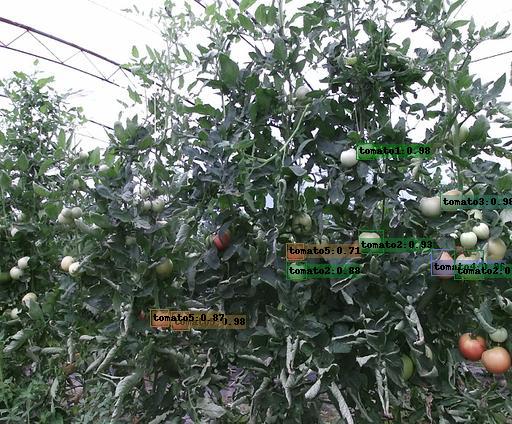

Supplement: Supplemental Information 1 [file peerj-cs-08-1018-s001.zip › data/0639.jpg]

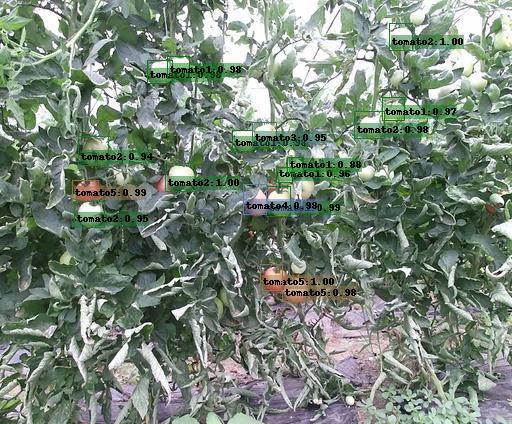

Supplement: Supplemental Information 1 [file peerj-cs-08-1018-s001.zip › data/0669.jpg]

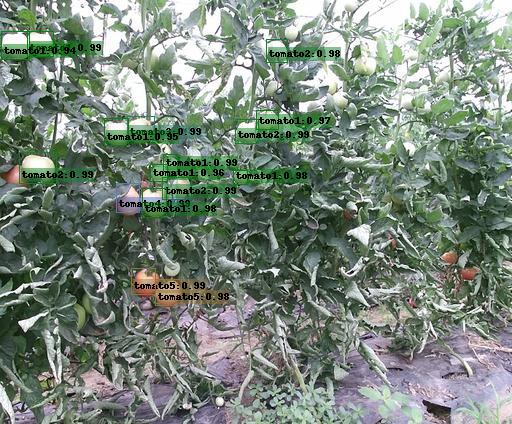

Supplement: Supplemental Information 1 [file peerj-cs-08-1018-s001.zip › data/0676.jpg]

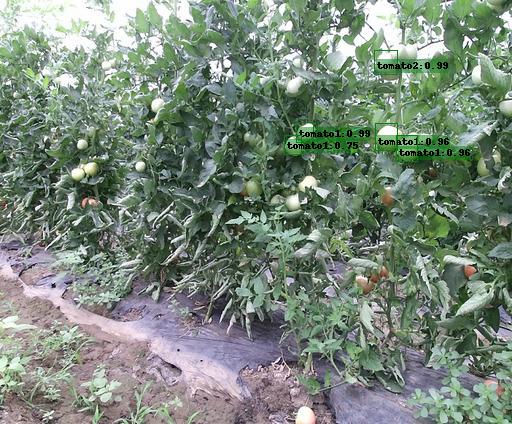

Supplement: Supplemental Information 1 [file peerj-cs-08-1018-s001.zip › data/0690.jpg]

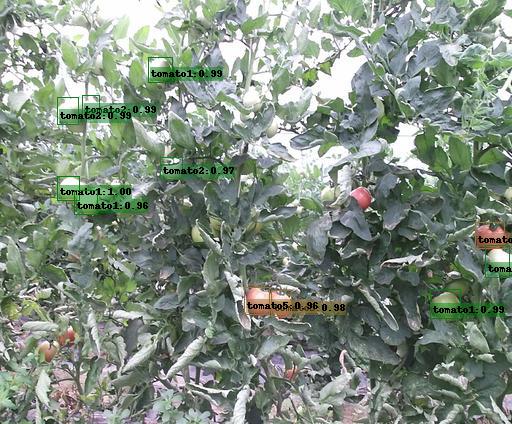

Supplement: Supplemental Information 1 [file peerj-cs-08-1018-s001.zip › data/0717.jpg]

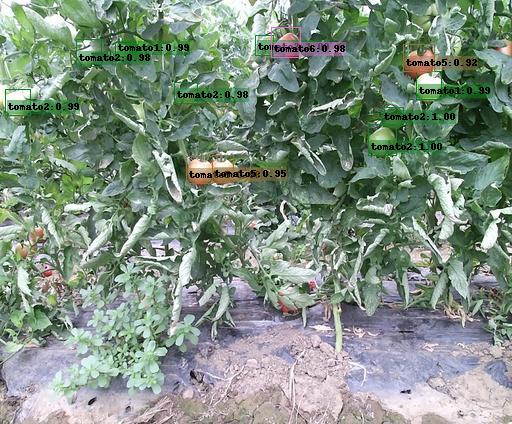

Supplement: Supplemental Information 1 [file peerj-cs-08-1018-s001.zip › data/0731.jpg]

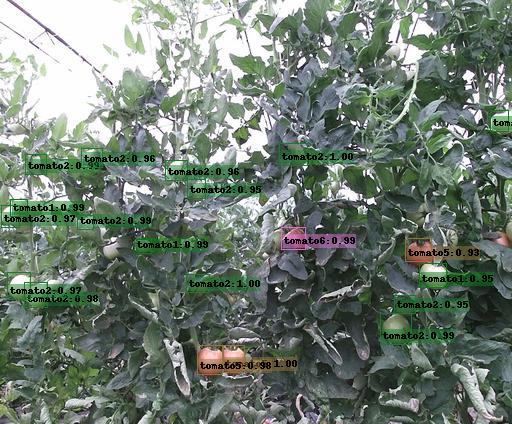

Supplement: Supplemental Information 1 [file peerj-cs-08-1018-s001.zip › data/0732.jpg]

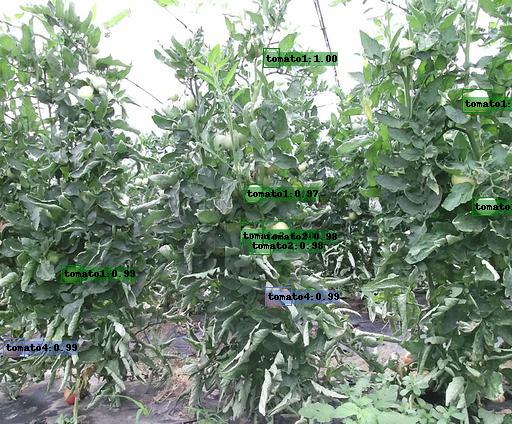

Supplement: Supplemental Information 1 [file peerj-cs-08-1018-s001.zip › data/0739.jpg]

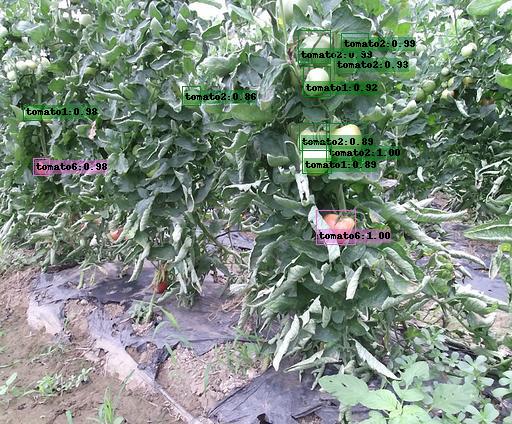

Supplement: Supplemental Information 1 [file peerj-cs-08-1018-s001.zip › data/0769.jpg]

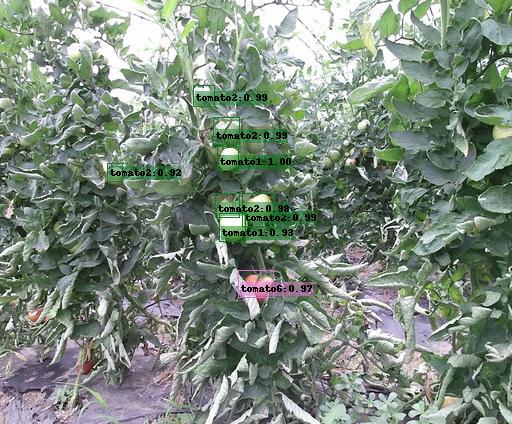

Supplement: Supplemental Information 1 [file peerj-cs-08-1018-s001.zip › data/0770.jpg]

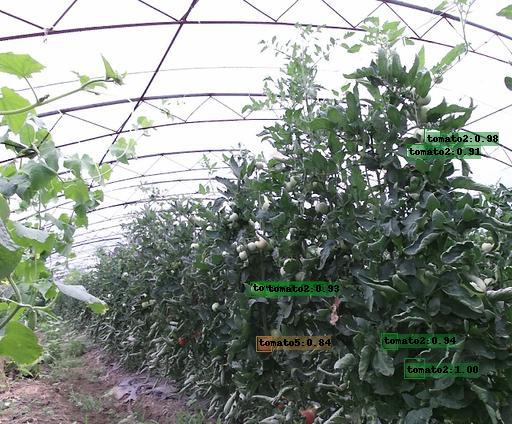

Supplement: Supplemental Information 1 [file peerj-cs-08-1018-s001.zip › data/0776.jpg]

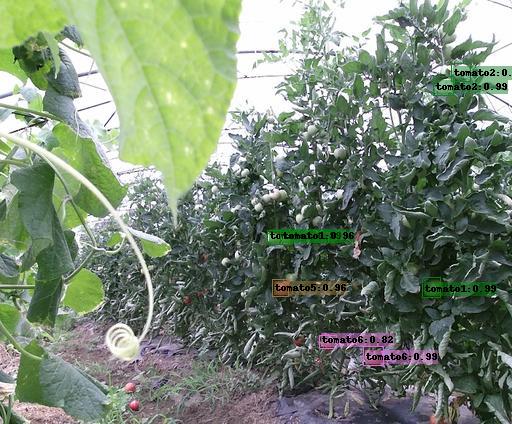

Supplement: Supplemental Information 1 [file peerj-cs-08-1018-s001.zip › data/0778.jpg]

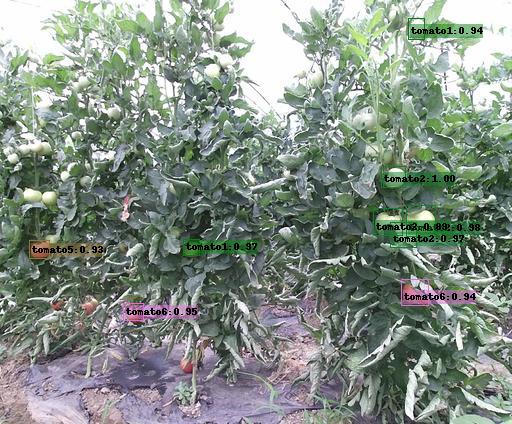

Supplement: Supplemental Information 1 [file peerj-cs-08-1018-s001.zip › data/0782.jpg]

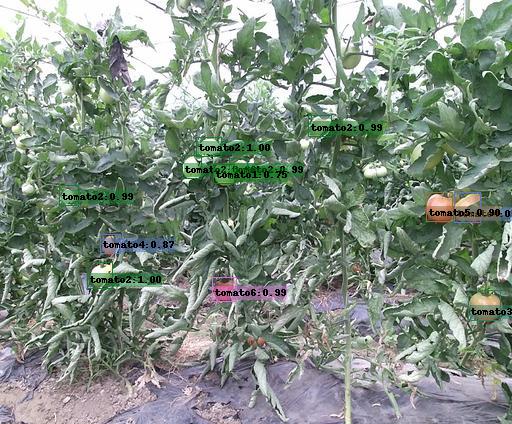

Supplement: Supplemental Information 1 [file peerj-cs-08-1018-s001.zip › data/0791.jpg]

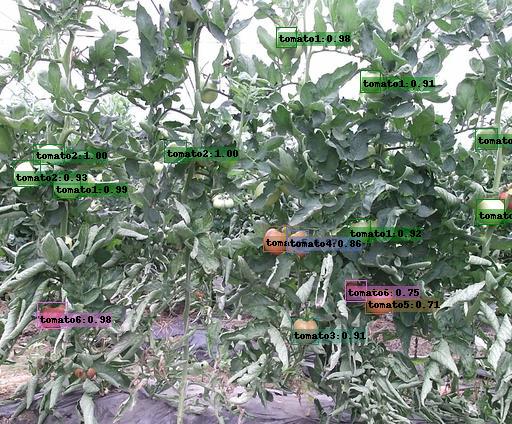

Supplement: Supplemental Information 1 [file peerj-cs-08-1018-s001.zip › data/0799.jpg]

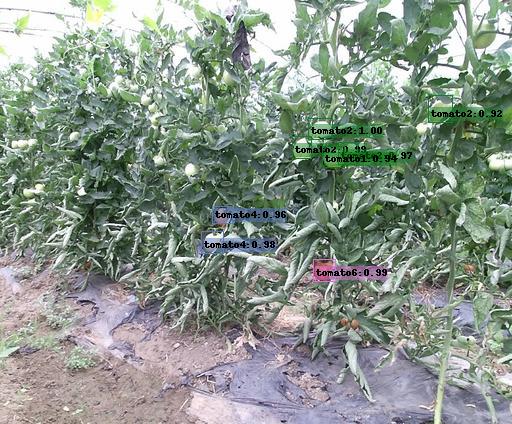

Supplement: Supplemental Information 1 [file peerj-cs-08-1018-s001.zip › data/0815.jpg]

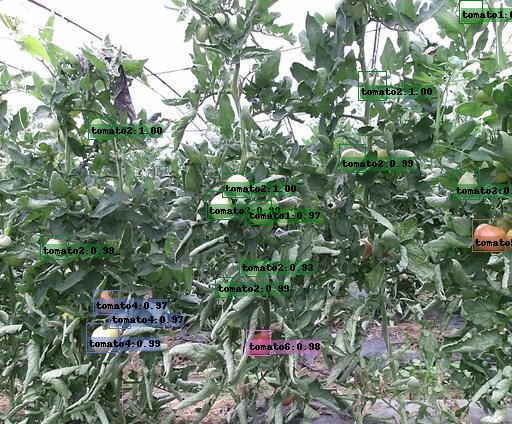

Supplement: Supplemental Information 1 [file peerj-cs-08-1018-s001.zip › data/0831.jpg]

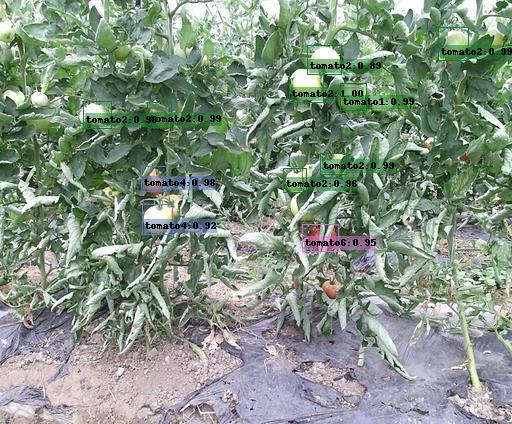

Supplement: Supplemental Information 1 [file peerj-cs-08-1018-s001.zip › data/0848.jpg]

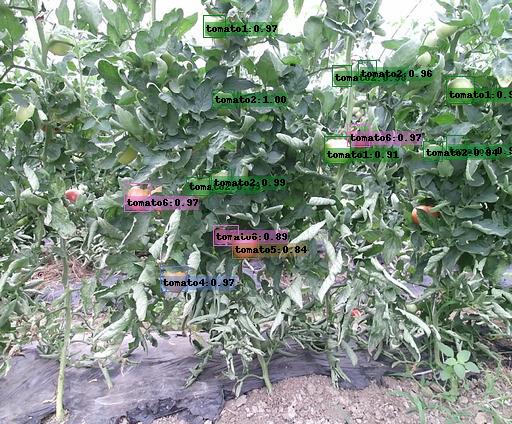

Supplement: Supplemental Information 1 [file peerj-cs-08-1018-s001.zip › data/0857.jpg]

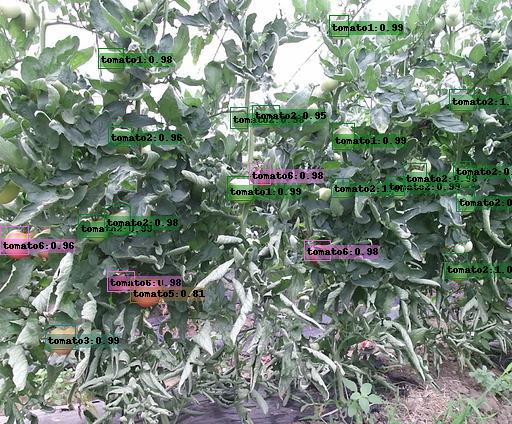

Supplement: Supplemental Information 1 [file peerj-cs-08-1018-s001.zip › data/0870.jpg]

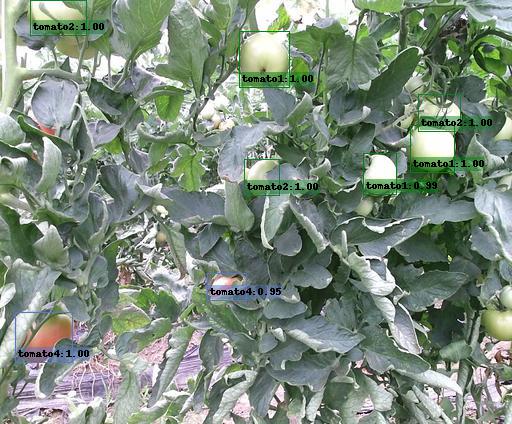

Supplement: Supplemental Information 1 [file peerj-cs-08-1018-s001.zip › data/0880.jpg]

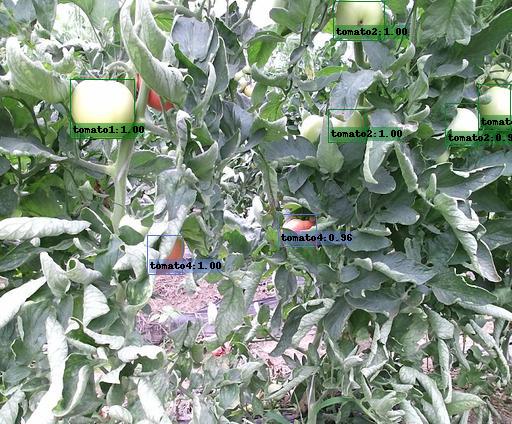

Supplement: Supplemental Information 1 [file peerj-cs-08-1018-s001.zip › data/0882.jpg]

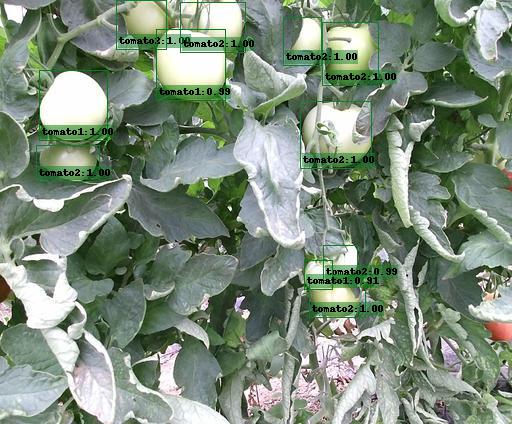

Supplement: Supplemental Information 1 [file peerj-cs-08-1018-s001.zip › data/0886.jpg]

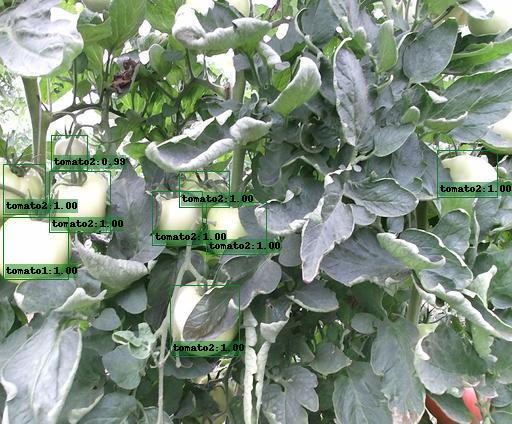

Supplement: Supplemental Information 1 [file peerj-cs-08-1018-s001.zip › data/0887.jpg]

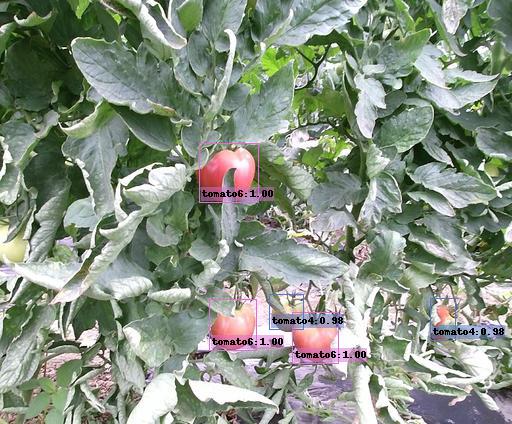

Supplement: Supplemental Information 1 [file peerj-cs-08-1018-s001.zip › data/0888.jpg]

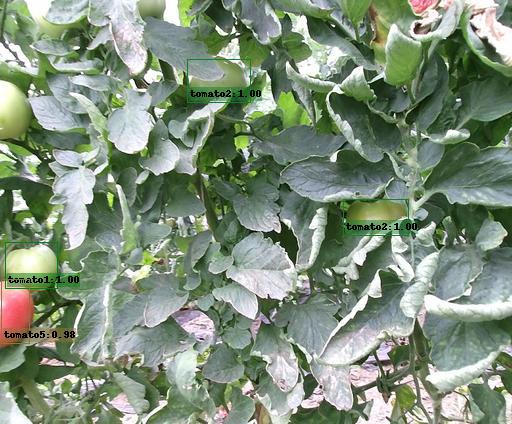

Supplement: Supplemental Information 1 [file peerj-cs-08-1018-s001.zip › data/0900.jpg]

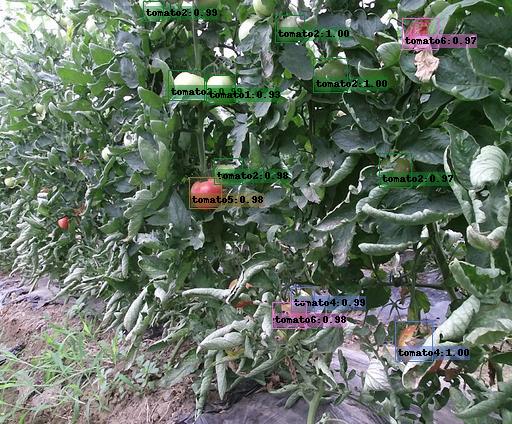

Supplement: Supplemental Information 1 [file peerj-cs-08-1018-s001.zip › data/0901.jpg]

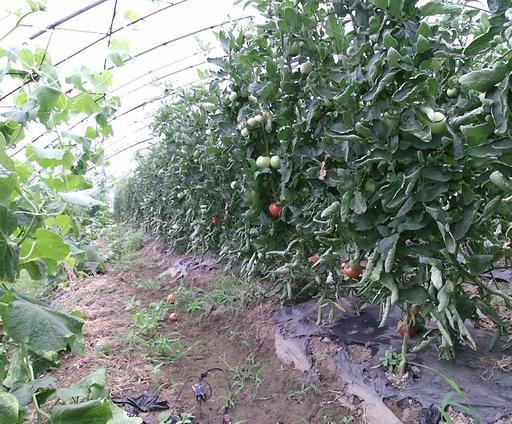

Supplement: Supplemental Information 1 [file peerj-cs-08-1018-s001.zip › data/0926.jpg]

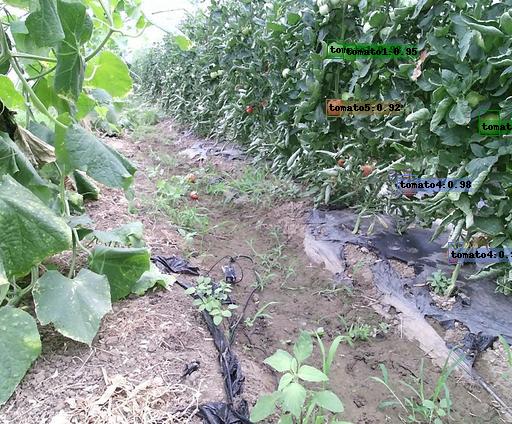

Supplement: Supplemental Information 1 [file peerj-cs-08-1018-s001.zip › data/0929.jpg]

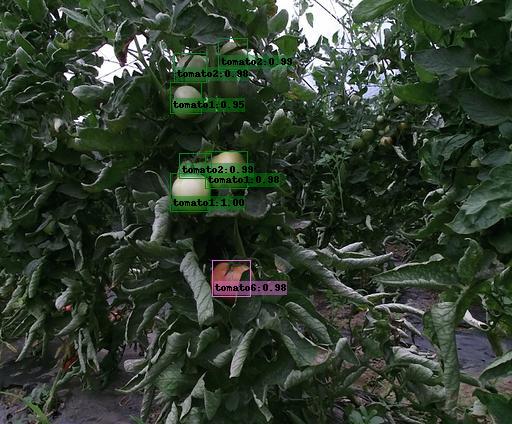

Supplement: Supplemental Information 1 [file peerj-cs-08-1018-s001.zip › data/0941.jpg]

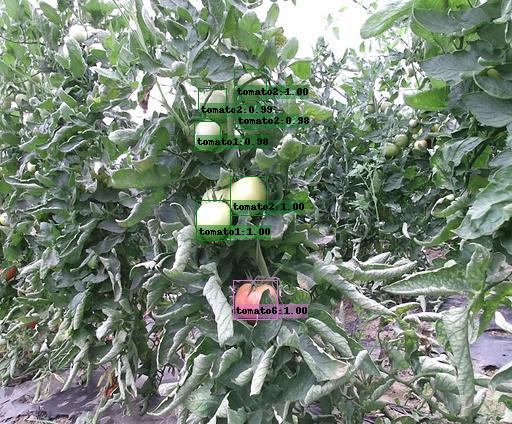

Supplement: Supplemental Information 1 [file peerj-cs-08-1018-s001.zip › data/0942.jpg]

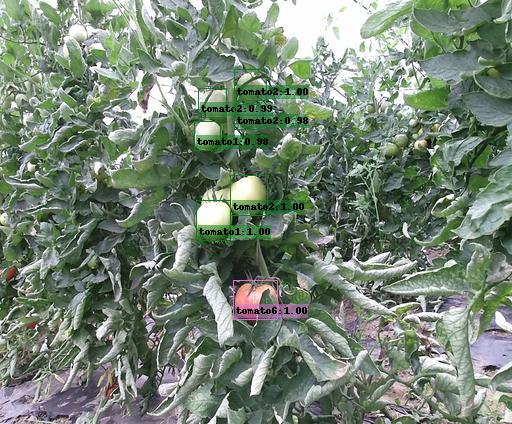

Supplement: Supplemental Information 1 [file peerj-cs-08-1018-s001.zip › data/0943.jpg]

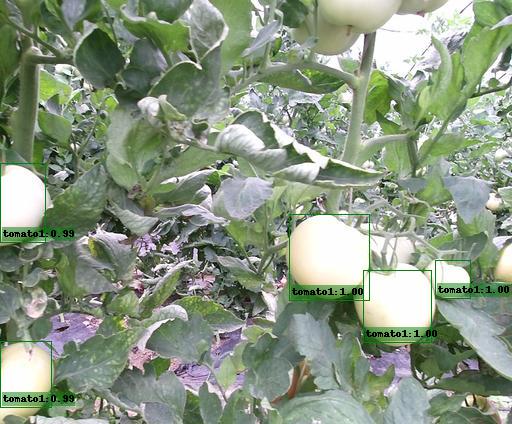

Supplement: Supplemental Information 1 [file peerj-cs-08-1018-s001.zip › data/0949.jpg]

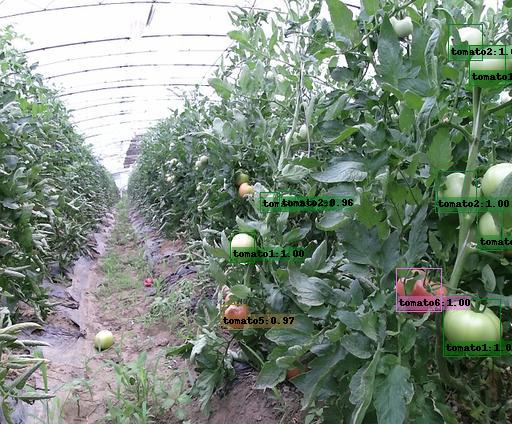

Supplement: Supplemental Information 1 [file peerj-cs-08-1018-s001.zip › data/1020.jpg]

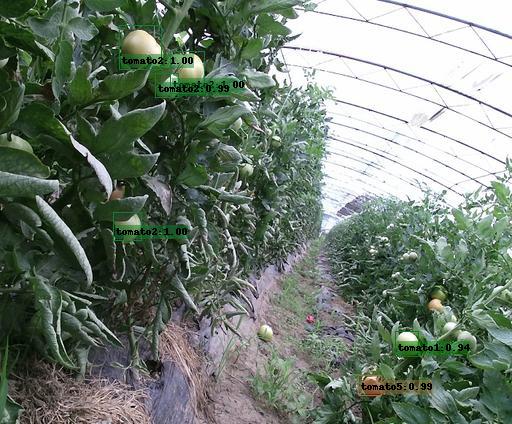

Supplement: Supplemental Information 1 [file peerj-cs-08-1018-s001.zip › data/1022.jpg]

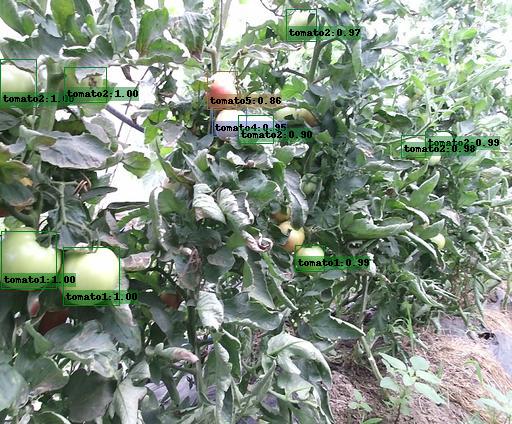

Supplement: Supplemental Information 1 [file peerj-cs-08-1018-s001.zip › data/1025.jpg]

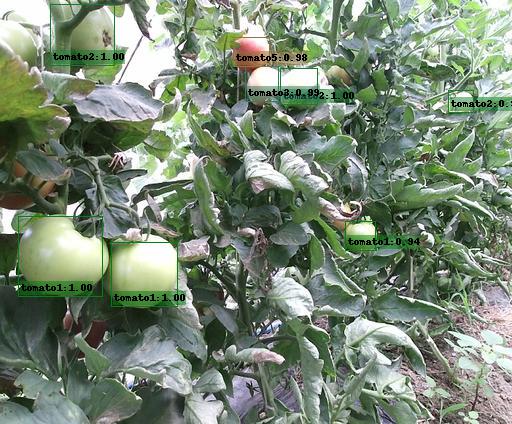

Supplement: Supplemental Information 1 [file peerj-cs-08-1018-s001.zip › data/1026.jpg]

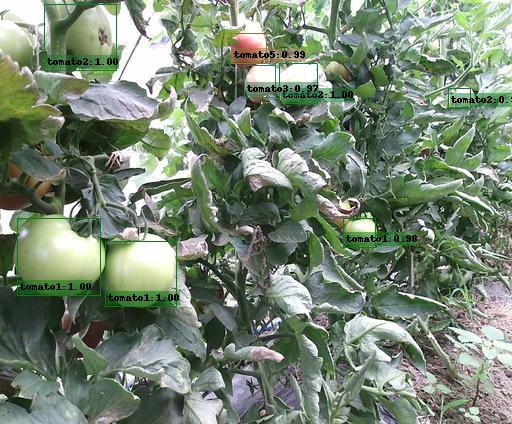

Supplement: Supplemental Information 1 [file peerj-cs-08-1018-s001.zip › data/1031.jpg]

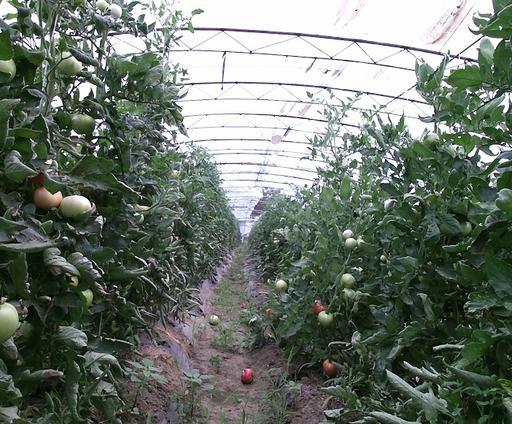

Supplement: Supplemental Information 1 [file peerj-cs-08-1018-s001.zip › data/1033.jpg]

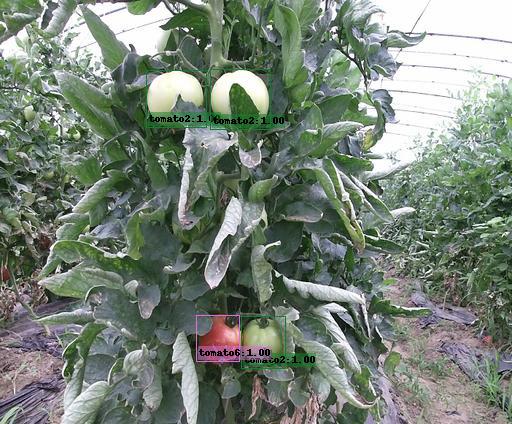

Supplement: Supplemental Information 1 [file peerj-cs-08-1018-s001.zip › data/1037.jpg]

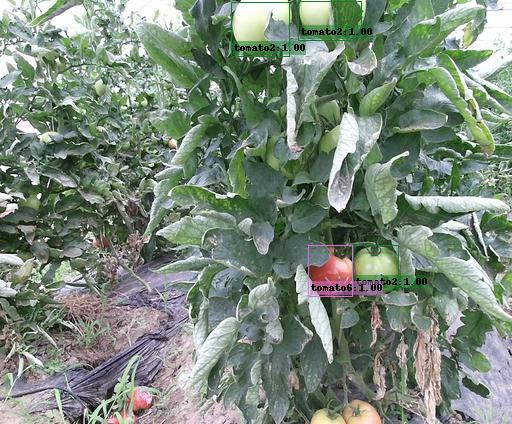

Supplement: Supplemental Information 1 [file peerj-cs-08-1018-s001.zip › data/1038.jpg]

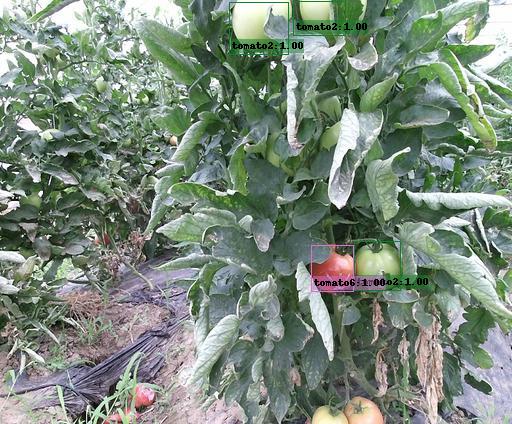

Supplement: Supplemental Information 1 [file peerj-cs-08-1018-s001.zip › data/1041.jpg]

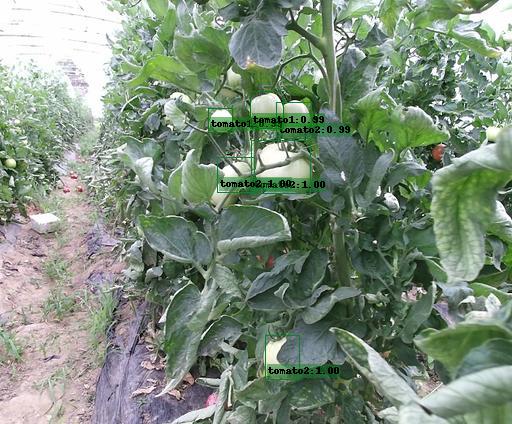

Supplement: Supplemental Information 1 [file peerj-cs-08-1018-s001.zip › data/1057.jpg]

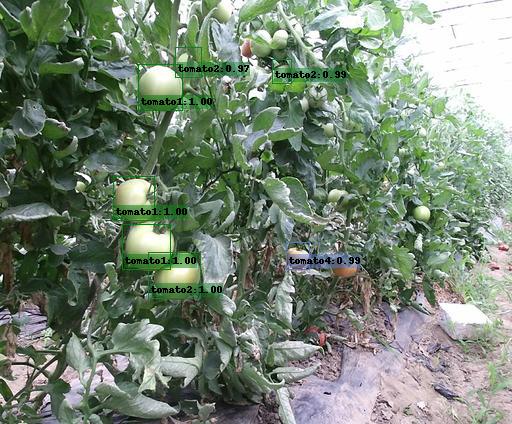

Supplement: Supplemental Information 1 [file peerj-cs-08-1018-s001.zip › data/1070.jpg]

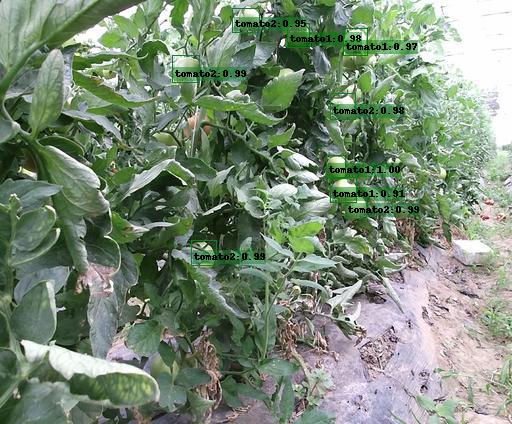

Supplement: Supplemental Information 1 [file peerj-cs-08-1018-s001.zip › data/1075.jpg]

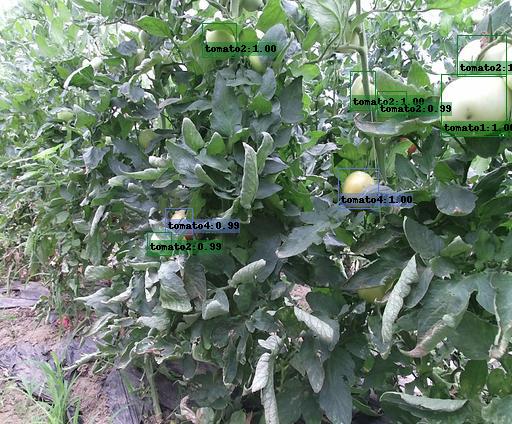

Supplement: Supplemental Information 1 [file peerj-cs-08-1018-s001.zip › data/1084.jpg]

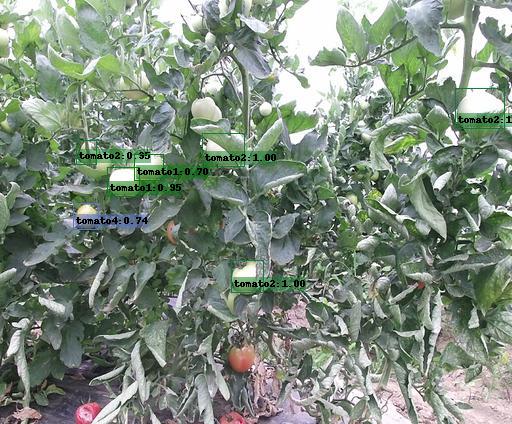

Supplement: Supplemental Information 1 [file peerj-cs-08-1018-s001.zip › data/1090.jpg]

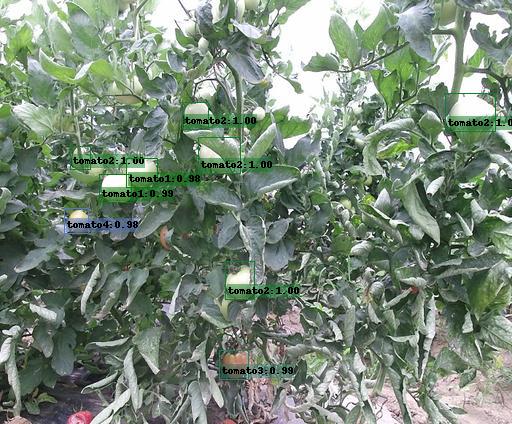

Supplement: Supplemental Information 1 [file peerj-cs-08-1018-s001.zip › data/1096.jpg]

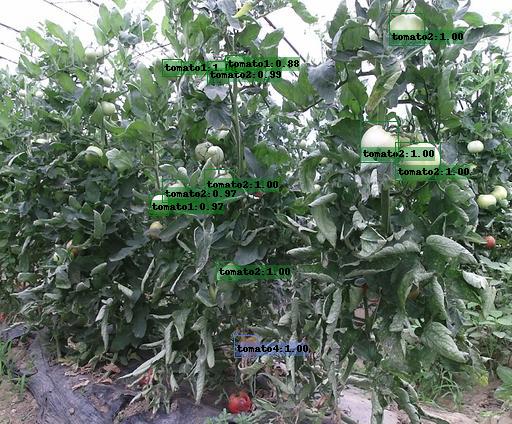

Supplement: Supplemental Information 1 [file peerj-cs-08-1018-s001.zip › data/1102.jpg]

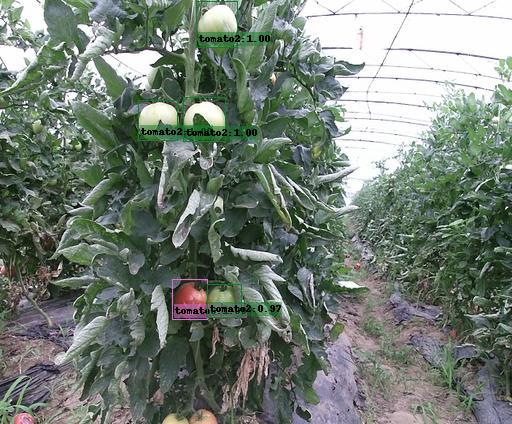

Supplement: Supplemental Information 1 [file peerj-cs-08-1018-s001.zip › data/1113.jpg]

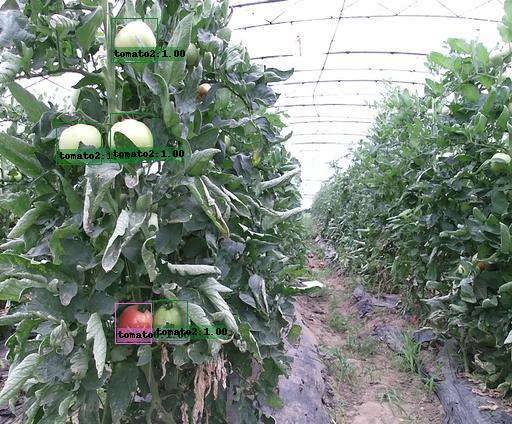

Supplement: Supplemental Information 1 [file peerj-cs-08-1018-s001.zip › data/1114.jpg]

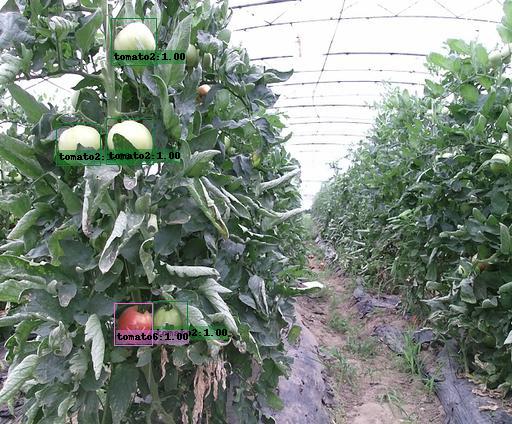

Supplement: Supplemental Information 1 [file peerj-cs-08-1018-s001.zip › data/1115.jpg]

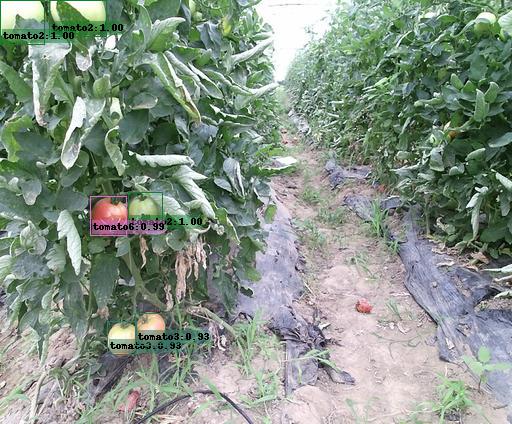

Supplement: Supplemental Information 1 [file peerj-cs-08-1018-s001.zip › data/1120.jpg]

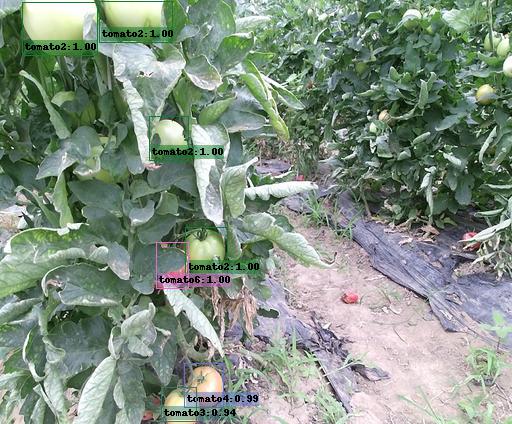

Supplement: Supplemental Information 1 [file peerj-cs-08-1018-s001.zip › data/1121.jpg]

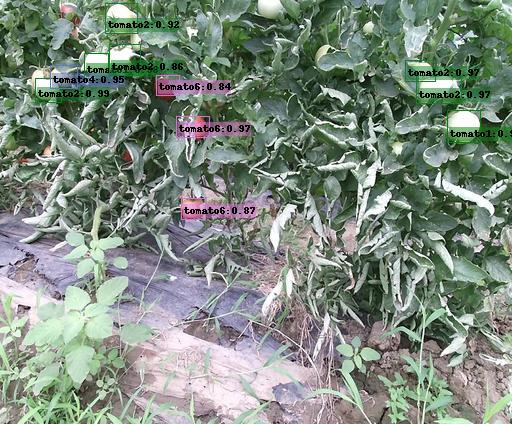

Supplement: Supplemental Information 1 [file peerj-cs-08-1018-s001.zip › data/1137.jpg]

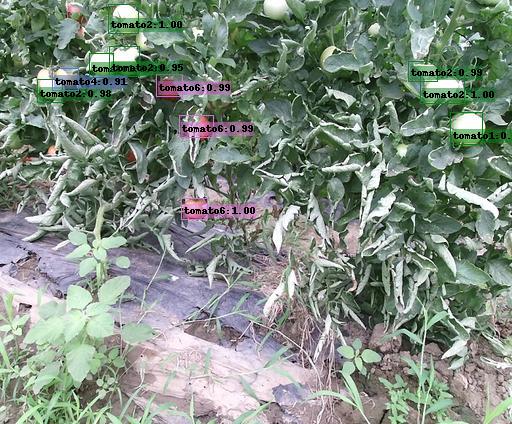

Supplement: Supplemental Information 1 [file peerj-cs-08-1018-s001.zip › data/1147.jpg]

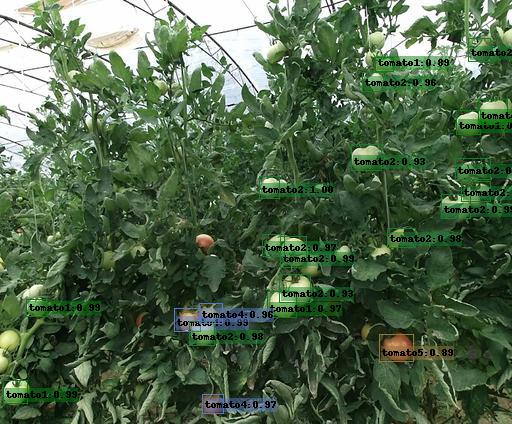

Supplement: Supplemental Information 1 [file peerj-cs-08-1018-s001.zip › data/1148.jpg]

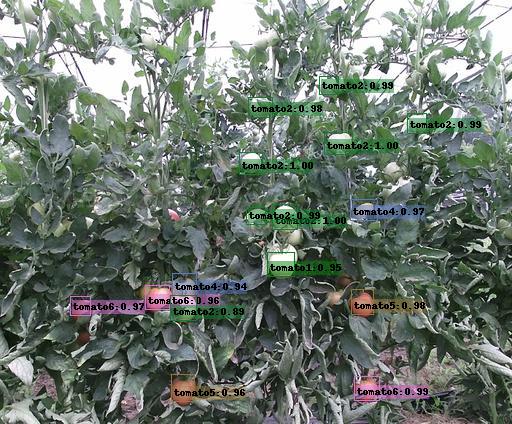

Supplement: Supplemental Information 1 [file peerj-cs-08-1018-s001.zip › data/1162.jpg]

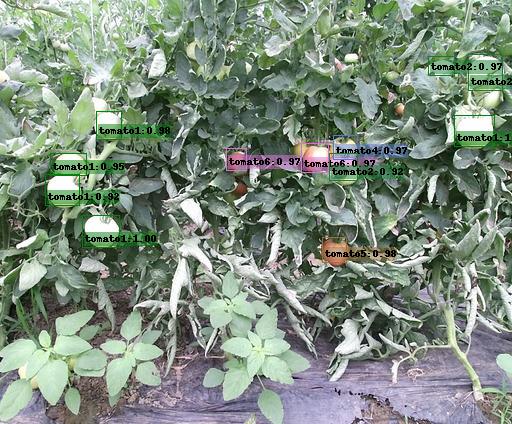

Supplement: Supplemental Information 1 [file peerj-cs-08-1018-s001.zip › data/1174.jpg]
